# CRTC Potentiates Light-independent *timeless* Transcription to Sustain Circadian Rhythms in *Drosophila*

**DOI:** 10.1038/srep32113

**Published:** 2016-08-31

**Authors:** Minkyung Kim, Hoyeon Lee, Jin-Hoe Hur, Joonho Choe, Chunghun Lim

**Affiliations:** 1 Department of Biological Sciences, Korea Advanced Institute of Science and Technology, Daejeon 34141, Republic of Korea; 2 School of Life Sciences, Ulsan National Institute of Science and Technology (UNIST), Ulsan 44919, Republic of Korea; 3 UNIST-Olympus Biomed Imaging Center (UOBC), UNIST, Ulsan 44919, Republic of Korea

## Abstract

Light is one of the strongest environmental time cues for entraining endogenous circadian rhythms. Emerging evidence indicates that CREB-regulated transcription co-activator 1 (CRTC1) is a key player in this pathway, stimulating light-induced *Period1* (*Per1*) transcription in mammalian clocks. Here, we demonstrate a light-independent role of *Drosophila* CRTC in sustaining circadian behaviors. Genomic deletion of the *crtc* locus causes long but poor locomotor rhythms in constant darkness. Overexpression or RNA interference-mediated depletion of CRTC in circadian pacemaker neurons similarly impairs the free-running behavioral rhythms, implying that *Drosophila* clocks are sensitive to the dosage of CRTC. The *crtc* null mutation delays the overall phase of circadian gene expression yet it remarkably dampens light-independent oscillations of TIMELESS (TIM) proteins in the clock neurons. In fact, CRTC overexpression enhances CLOCK/CYCLE (CLK/CYC)-activated transcription from *tim* but not *per* promoter in clock-less S2 cells whereas CRTC depletion suppresses it. Consistently, TIM overexpression partially but significantly rescues the behavioral rhythms in *crtc* mutants. Taken together, our data suggest that CRTC is a novel co-activator for the CLK/CYC-activated *tim* transcription to coordinate molecular rhythms with circadian behaviors over a 24-hour time-scale. We thus propose that CRTC-dependent clock mechanisms have co-evolved with selective clock genes among different species.

Most living organisms have evolved endogenous time-keeping mechanisms known as circadian clocks to anticipate and adapt to daily changes in the environment. External time cues, such as cycles of light, temperature or food availability, entrain the circadian oscillators to sustain 24-hour rhythms. Timing information is subsequently translated into other physiological pathways of the organism, such as sleep, metabolism, immune responses and so forth^1^,^2^,^3^.

At the molecular level, a transcriptional feedback network of circadian transcription factors that regulates daily rhythmic gene expression constitutes a basic framework for cell-autonomous molecular clocks^4^. In *Drosophila*, CLK-CYC, a heterodimeric transcription factor composed of CLOCK and CYCLE, binds E-box sequences and activates the transcription of *period* (*per*) and *timeless* (*tim*) genes from the late afternoon to midnight. PER-TIM complexes accumulate in the cytoplasm at early night and are then translocated to the nucleus, where they inhibit the transcriptional activity of CLK-CYC after midnight. In the morning, light-dependent TIM degradation and cumulative PER phosphorylation destabilize the PER-TIM complex. The consequent de-repression of CLK-CYC activity leads to a new daily cycle^5^,^6^,^7^,^8^,^9^. Additional layers of transcriptional feedback loops contribute to the robustness of the molecular clockwork, maintaining high-amplitude rhythms and diverse clock outputs^10^,^11^,^12^,^13^,^14^. Moreover, key players in the negative feedback loops are homologous or functionally analogous between flies and mammals^15^,^16^,^17^.

Light stimulates mammalian *Per* expression via CREB (cAMP response element binding protein)-dependent transcriptional activation, playing important roles in the photic entrainment of mammalian clocks^18^,^19^,^20^. Recent studies have shown that this process requires CRTC (CREB-regulated transcription co-activator)^21^. In fact, CRTC and its negative regulator SIK1 (salt-inducible kinase 1) constitute a negative feedback loop. Light-activated CRTC induces *Sik1* transcription, and then elevated SIK1 feeds back to phosphorylate CRTC proteins, blocking their nuclear entry^22^,^23^,^24^. This mechanism buffers the light-dependent, phase-resetting of clocks such that animals are able to robustly sustain circadian rhythms rather than changing their circadian phase back and forth in response to sudden changes in light regime.

Here, we identify an unexpected role of CRTC in *Drosophila* clocks and demonstrate that *Drosophila* CRTC activates *tim* transcription to align circadian gene expression on a 24-hour time-scale and drive robust free-running rhythms in circadian behaviors. Light-independent effects of *Drosophila crtc* are evident in the molecular rhythms of both central pacemaker neurons and peripheral clock tissues, implicating an ancestral origin of CRTC-dependent clocks. Given distinct clock functions of CRTC homologs, we suggest a model on how CRTC-dependent clock mechanisms have co-evolved with selective clock targets among different species.

## Results

### *crtc* mutation causes long but poor rhythms in circadian behaviors

**Figure 1:**
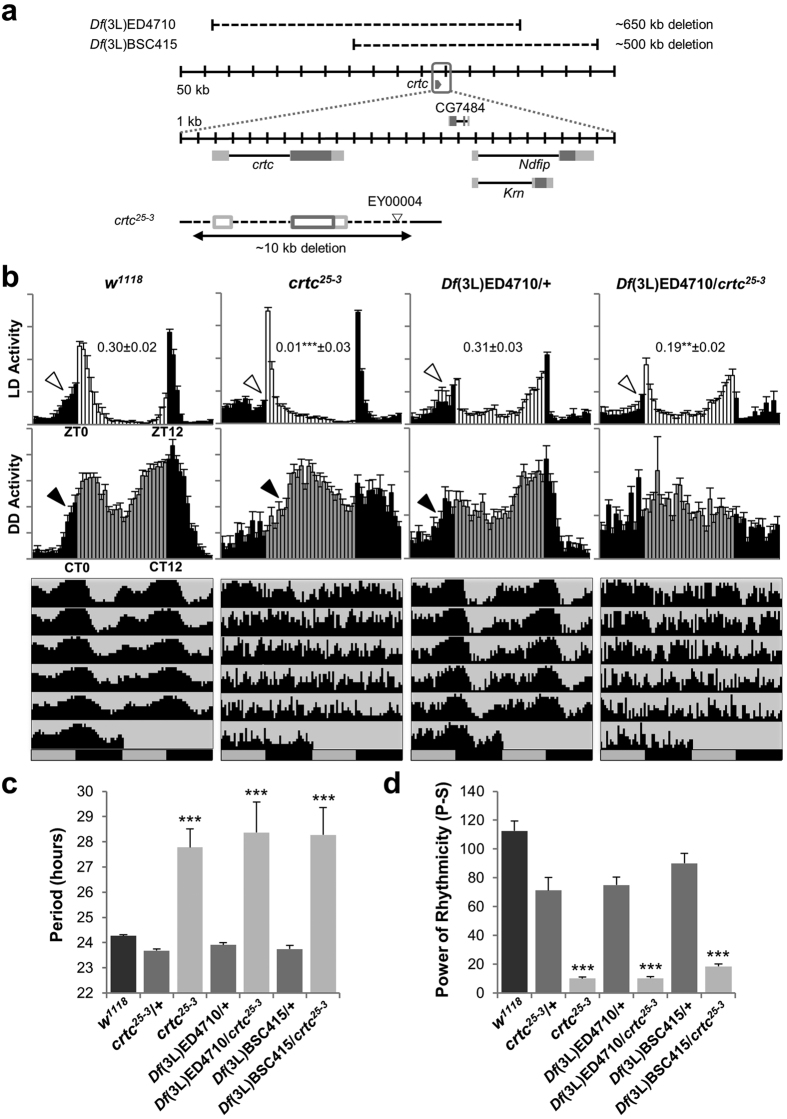
*Drosophila* CRTC is necessary for robust circadian behaviors. (**a**) A schematic diagram of *crtc* mutant alleles. Dark-gray boxes, the translated regions of exons; light-gray boxes, untranslated regions. (**b**) Male flies homozygous or trans-heterozygous for *crtc* mutant alleles show long but poor rhythms in circadian behaviors. Normalized activity profiles in LD cycles (top) or on the first day of DD cycles (middle) were averaged from individual flies. Averaged actograms throughout the behavioral analyses were double-plotted (bottom). Anticipatory increase in locomotor activities prior to lights-on (i.e., morning anticipation) was quantified by calculating morning index in individual flies as described in Materials and Methods. Averaged morning index values +/− SEM were shown in the LD activity profiles. White arrow heads, morning anticipation in LD cycles; black arrow heads, morning anticipation in the first DD cycle; white/black bars, LD cycles; gray/black bars, DD cycles; ZT, zeitgeber time; CT, circadian time. Error bars indicate SEM. (**c**) Circadian periods in DD locomotor rhythms were averaged from rhythmic flies (*P*-*S* > 10; see below). ***P < 0.001 to wild-type (*w*^*1118*^) and all heterozygous controls as determined by one-way ANOVA, Tukey post hoc test. Error bars indicate SEM. (**d**) Rhythmicity in free-running locomotor behaviors was determined by measuring power (*P*) - significance (*S*) values from the chi-squared periodograms of individual flies and averaged per each genotype. ***P < 0.001 to wild-type (*w*^*1118*^) and all heterozygous controls as determined by one-way ANOVA, Tukey post hoc test. Error bars indicate SEM.

To determine if CRTC regulates circadian rhythms in *Drosophila*, we first examined how CRTC loss-of-function affects circadian behaviors. We tested a *crtc*^*25-3*^ allele lacking the entire *crtc* locus as a result of imprecise excision of a transposable element insert (Fig. 1a)^25^. Wild-type flies showed bimodal peaks of locomotor activity in light: dark (LD) cycles of 12 hours on and 12 hours off (Fig. 1b). They also anticipated the timing of lights-on and -off by gradually increasing locomotor activity around the light transitions. In contrast, *crtc* mutants displayed compromised morning anticipation as supported by their lower morning index (Fig. 1b, top). A quantitative assessment of circadian periods and rhythmicity in constant dark (DD) following LD entrainment revealed that *crtc* mutants largely exhibited arrhythmic behaviors with rapid dampening of free-running activity peaks (Fig. 1b–d, Supplementary Table 1). Nonetheless, *crtc* mutants with detectable rhythmicity showed long-period rhythms (Fig. 1c, Supplementary Table 1, Supplementary Fig. 1). Consistently, we observed a phase delay in the anticipatory morning activity peak of *crtc* mutants in the first DD cycle (Fig. 1b, middle), suggesting that their morning anticipation in LD cycles was actually masked by a startling response to lights-on. We also noticed that 27% of *crtc* mutants died during our behavioral tests whereas the majority of control flies survived (Supplementary Fig. 2a). Since *crtc* mutants are more sensitive to starvation^25^, we reasoned that 5% sucrose food used in our behavioral tests might partially mimic starvation conditions, thereby lowering the survival rate in *crtc* mutants. Behavioral tests on 10% sucrose or corn-meal food indeed rescued the lower survival rate in *crtc* mutants yet they still displayed the poor rhythmicity on the enriched food (Supplementary Fig. 2b, Supplementary Table 2). It is thus unlikely that *crtc* mutants lose behavioral rhythmicity as they become starved and sick. Finally, chromosomal deletions covering the *crtc* locus did not complement these circadian defects in trans-heterozygotes with *crtc*^*25-3*^ allele (Fig. 1b–d, Supplementary Table 1), further supporting that CRTC is necessary for sustaining robust circadian behaviors in *Drosophila*.

### *Drosophila* clocks are sensitive to the dosage of CRTC in PDF-expressing neurons

**Figure 2:**
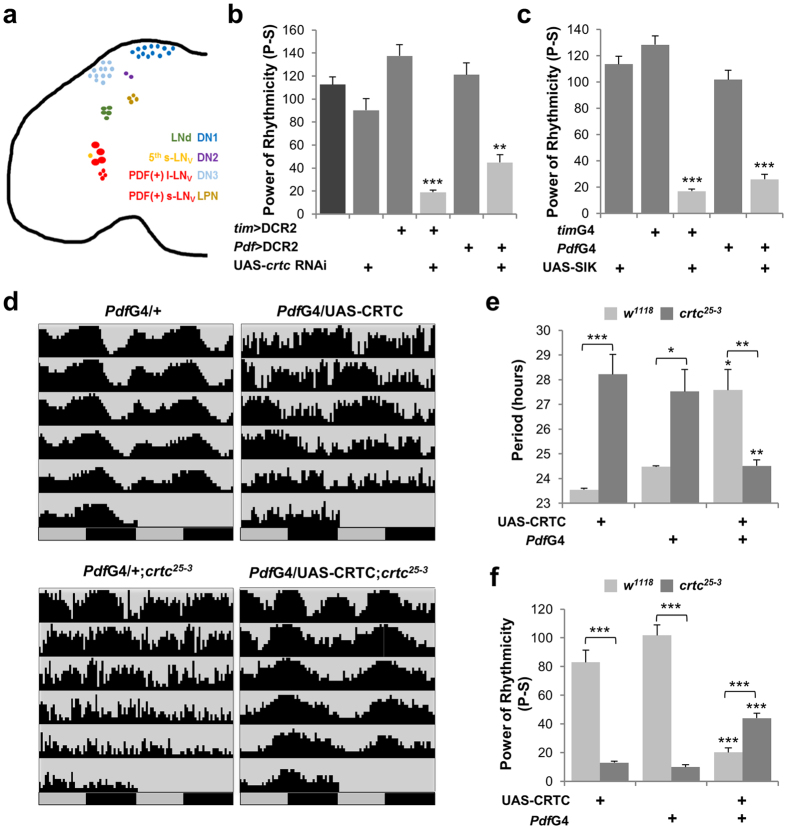
*Drosophila* clocks are sensitive to the dosage of CRTC in PDF Neurons. (**a**) Circadian pacemaker neurons in adult fly brain. *tim*-Gal4 is expressed in all clock neurons covering lateral neurons (LNs), dorsal neurons (DNs), and lateral posterior neurons (LPNs). *Pdf*-Gal4 is expressed specifically in PDF-positive large and small ventral LNs (l-LNv and s-LNv, respectively). LNd, dorsal LN; 5^th^ s-LNv, a single s-LNv not expressing a PDF neuropeptide. (**b**) CRTC depletion in circadian pacemaker neurons leads to arrhythmic circadian behaviors. *crtc* RNAi transgene was co-expressed along with the RNAi-enhancing DCR2 by *tim*-Gal4 or *Pdf*-Gal4 driver. Rhythmicity in free-running locomotor behaviors in DD cycles was measured similarly as in Fig. 1d. **P < 0.01, ***P < 0.001 to controls heterozygous for Gal4 > DCR2 or UAS-*crtc* RNAi as determined by one-way ANOVA, Tukey post hoc test. Error bars indicate SEM. (**c**) SIK2 overexpression in circadian pacemaker neurons by *tim*-Gal4 or *Pdf*-Gal4 driver leads to arrhythmic circadian behaviors. ***P < 0.001 to controls heterozygous for Gal4 or UAS-SIK as determined by one-way ANOVA, Tukey post hoc test. Error bars indicate SEM. (**d**) CRTC overexpression in PDF neurons rescues 24-hour behavioral rhythms in *crtc* mutants. Averaged actograms in DD cycles were double-plotted with their genotypes on top. (**e**,**f**) Circadian periods and rhythmicity in DD locomotor behaviors were measured as above. *P < 0.05, **P < 0.01 and ***P < 0.001 to controls heterozygous for *Pdf*-Gal4 or UAS-CRTC in wild-type (light-gray bars) or *crtc* mutants (dark-gray bars) as determined by one-way ANOVA, Tukey post hoc test. Error bars indicate SEM.

There are ~150 circadian pacemaker neurons that exhibit daily circadian gene expression in the adult fly brain (Fig. 2a). Based on their anatomical location and specific clock gene expression, these clock neurons can be divided into distinct groups that govern different aspects of circadian behaviors^26^,^27^,^28^,^29^. To map neural loci important for CRTC-dependent circadian behaviors, we examined whether CRTC depletion in specific neurons affected free-running locomotor behaviors. We first validated that our *crtc* RNA interference (RNAi) transgene decreased endogenous levels of CRTC proteins in fly heads by 30~60% when overexpressed by the pan-neuronal *Elav*-Gal4 driver or by the clock cell-specific *tim*-Gal4 driver (Supplementary Fig. 3). As expected, CRTC depletion in all canonical pacemaker neurons led to long but poor behavioral rhythms, phenocopying *crtc* mutants (Fig. 2b, Supplementary Table 3, *tim* > DCR2, *crtc* RNAi). When CRTC depletion was more restricted to large and small ventral lateral neurons (LNv) expressing the circadian neuropeptide PDF (pigment-dispersing factor) (referred to as PDF neurons hereafter)^30^,^31^,^32^, modest but significant decrease in DD rhythmicity was observed as compared to control flies (Fig. 2b, Supplementary Table 3, *Pdf* > DCR2, *crtc* RNAi). Consistent with the RNAi phenotype, PDF neuron-specific overexpression of *Drosophila* SIK2, a negative regulator of CRTC^33^,^34^, lengthened circadian periods and dampened behavioral rhythms in DD (Fig. 2c, Supplementary Table 4).

To determine whether CRTC expression in PDF neurons would be sufficient for robust 24-hour rhythms in DD, we overexpressed CRTC in PDF neurons of *crtc* mutant flies and compared their circadian behaviors to those in transgenic controls. Indeed, PDF neuron-specific CRTC overexpression restored 24-hour periodicity in circadian behaviors of *crtc* mutants whereas it partially but significantly rescued the rhythmicity phenotype (Fig. 2d–f, Supplementary Fig. 1, Supplementary Table 5). Unexpectedly, we also found that CRTC overexpression in PDF neurons caused long and weak behavioral rhythms in wild-type flies. These data indicate the presence of a window of CRTC levels or activities effective for the proper control of circadian behaviors, possibly explaining the partial rescue in *crtc* mutants.

### Conditional manipulation of CRTC expression in PDF neurons of adult flies is sufficient to affect circadian behaviors

To understand how the genetic manipulation of *crtc* in PDF neurons affects circadian behaviors, we first tested if *crtc* mutant flies normally develop circadian pacemaker neurons important for behavioral rhythms. CRTC-depleted or CRTC-overexpressing flies did not show any gross defects in the cell bodies or axonal projections of their PDF neurons (Supplementary Fig. 4). However, *crtc* mutants constitutively exhibited a higher degree of the axonal arborizations from their small LNv and stronger intensities for anti-PDF staining in the dorsal projections whereas control flies showed rhythmic arborizations and cycling PDF levels in the axonal termini of the pacemaker neurons in LD cycles^35^,^36^ (Supplementary Fig. 5). Of note, similar phenotypes have been reported in clock-less *tim* null mutant flies although *tim* mutants have more but shorter PDF branches than control flies^35^,^36^,^37^. To further exclude the possibility of *crtc* effects on the development of PDF neurons, we transiently overexpressed *crtc* RNAi or CRTC cDNA during the period of behavioral tests. To this end, we employed a *Pdf*-GeneSwitch-Gal4 driver, which is activated by feeding on mifepristone (RU486)-containing food but not on food containing ethanol (vehicle control), to drive transgenic expression in PDF neurons^38^. This conditional depletion or overexpression of CRTC in PDF neurons significantly decreased behavioral rhythmicity in DD while modest lengthening of circadian periods was observed in *crtc* RNAi flies (Supplementary Fig. 6, Supplementary Table 6). Taken together, these data convincingly demonstrate that *Drosophila* circadian behaviors are sensitive to CRTC levels in PDF neurons of adult fly brains.

### Circadian gene expression is phase-delayed by *crtc* mutation

**Figure 3:**
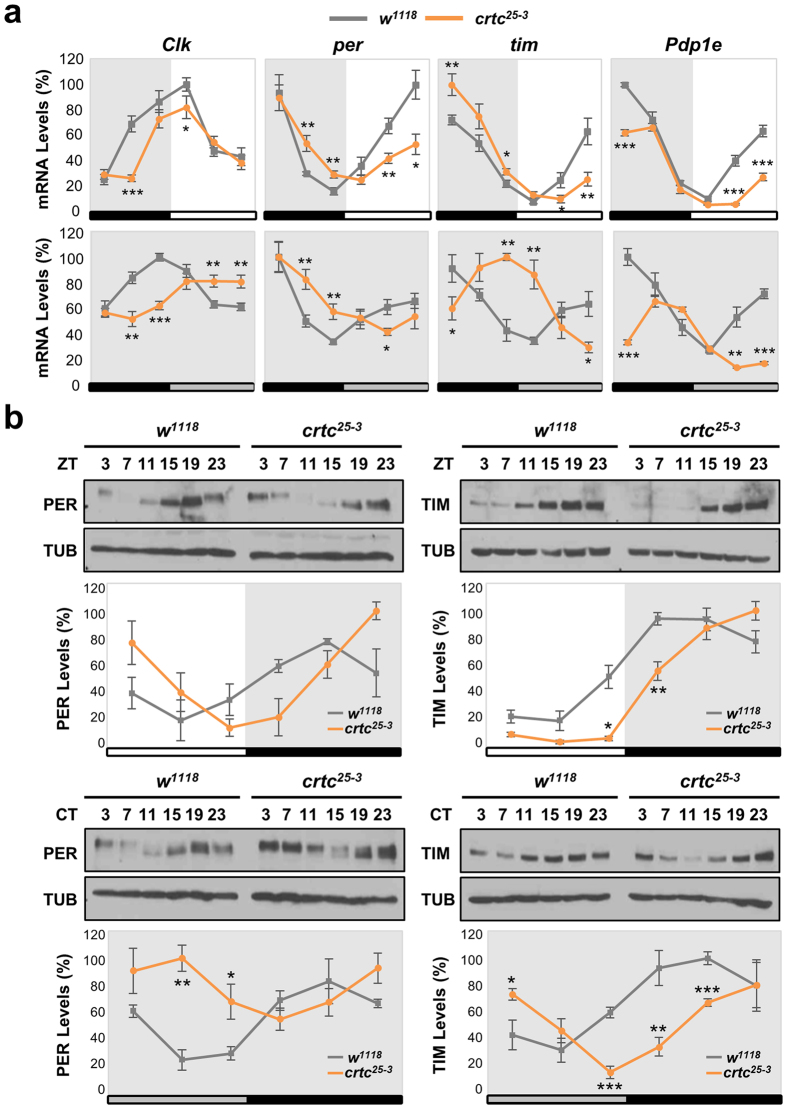
*crtc* mutation phase-delays circadian gene expression in adult fly heads. (**a**) Circadian expression of clock mRNAs in adult fly heads of wild-type (gray lines) and *crtc* mutants (orange lines). Flies were collected at six different time-points in LD cycles (top) or during the transition from the first to the second DD cycle following LD entrainment (bottom) and total RNAs from heads were purified. Relative levels of *Clk*, *per*, *tim*, and *Pdp1* mRNAs were quantified by real-time RT-PCR. X-axis indicates zeitgeber time (ZT) in LD (top) or circadian time (CT) in DD (bottom). Y-axis indicates relative expression levels (%) at each time-point, calculated by normalizing to the peak value (set as 100). White/black bars, LD cycles; gray/black bars, DD cycles. Data represent the average of three independent experiments. *P < 0.05, **P < 0.01 and ***P < 0.001 as determined by Student’s *t*-test. Error bars indicate SEM. (**b**) Circadian expression of PER and TIM proteins in adult fly heads of wild-type (gray lines) and *crtc* mutants (orange lines). Head extracts were prepared from flies harvested during LD (top) or the first DD (bottom) cycle and immunoblotted with anti-PER, anti-TIM and anti-TUBULIN (TUB, loading control) antibodies under the same experimental conditions. Images were cropped from full-length blots shown in Supplementary Fig. 12. Protein band intensities in each lane were quantified using ImageJ software and normalized to that of TUB protein. Y-axis indicates the relative expression levels (%) of PER and TIM proteins, calculated by normalizing to the peak value (set as 100). White/black bars, LD cycles; gray/black bars, DD cycles. Data represent the average of three independent experiments. *P < 0.05, **P < 0.01 and ***P < 0.001 as determined by Student’s *t*-test. Error bars indicate SEM.

Given the transcriptional co-activator function of CRTC^33^, we reasoned that CRTC-dependent transcription of clock-relevant genes may explain adult-specific roles of CRTC in sustaining 24-hour behavioral rhythms. We thus examined how CRTC loss-of-function affected the cycling expression of core clock genes in fly heads. Wild-type flies exhibited daily oscillations in abundance of clock mRNAs in both LD and DD cycles (Fig. 3a). The circadian phase of *Clk* mRNA rhythms was in anti-phase with those of its transcriptional targets *per*, *tim* and *Pdp1* (Par domain protein 1)^10^. We found that *crtc* mutants displayed a ~4-hour delay in the increasing phase of circadian mRNA expression in LD cycles while *crtc* effects on the declining phase were relatively weak (Fig. 3a, top). The phase delay in mRNA rhythms became more evident during the first-to-second DD cycles following LD cycles (Fig. 3a, bottom), when *crtc* mutant flies rapidly lost their behavioral rhythms. Accordingly, the circadian expression of PER and TIM proteins in fly head extracts was phase-delayed in *crtc* mutant flies (Fig. 3b). PER proteins are cumulatively phosphorylated for their ubiquitin-dependent degradation^39^,^40^. Wild-type flies thus displayed time-dependent PER phosphorylation in LD cycles, as assessed by shifts in gel mobility (Fig. 3b, top). In *crtc* mutants, PER phosphorylation was not readily observed until the late D phase of LD cycles, further validating the phase delay in molecular rhythms. *crtc* mutation had stronger impacts on the oscillating phase of PER and TIM in DD cycles (Fig. 3b, bottom), possibly due to lack of light-induced TIM degradation^41^,^42^.

We next asked if CRTC overexpression has opposing effects on circadian gene expression. That is, we reasoned that CRTC overexpression would activate the rate-limiting circadian transcription, thereby phase-advancing the molecular clocks. To our surprise, however, CRTC overexpression in *tim*-expressing clock neurons severely dampened the rhythmic amplitude or levels of all clock-relevant mRNAs tested in fly heads (Supplementary Fig. 7). While phase delays were evident in the low-amplitude rhythms of both *per* and *tim* mRNAs, non-cycling levels of PDF receptor mRNA decreased by ~50%. These inconsistent effects of CRTC loss-of-function versus CRTC overexpression suggest a possibility that a molar excess of free CRTC proteins might interfere with baseline transcriptional activities in the clock neurons of CRTC-overexpressing flies, likely through the sequestration of general transcription factors. In fact, we observed a similar phenomenon that the transcriptional activation of reporter genes harboring *per* or *tim* promoters is rather attenuated by highly overexpressed CLK (Supplementary Fig. 8).

### *crtc* mutation impacts on PER and TIM oscillations in circadian pacemaker neurons

**Figure 4:**
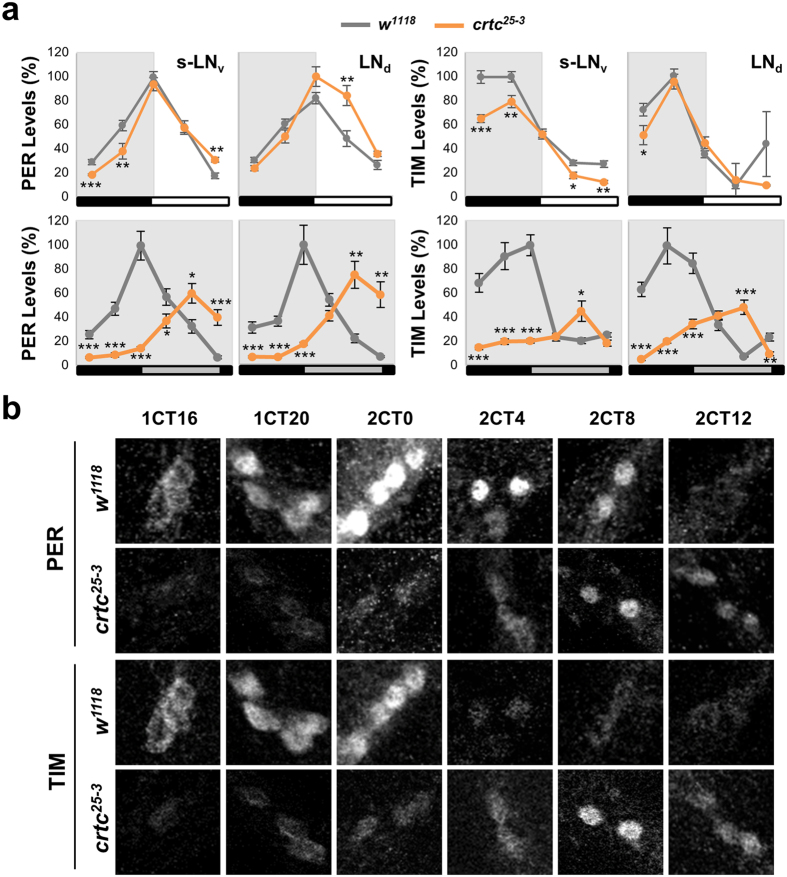
*crtc* mutation impacts on PER and TIM oscillations in circadian pacemaker neurons. (**a**) Circadian expression of PER and TIM proteins in circadian pacemaker neurons of wild-type (gray lines) and *crtc* mutants (orange lines). Adult fly brains were dissected at different time-points in LD cycles (top) or during the transition from the first to the second DD cycle following LD entrainment (bottom). Whole-mount immunostaining was performed using anti-PER, anti-TIM, and anti-PDF antibodies. Confocal brain images were obtained from 12–13 hemispheres at each time-point (X-axis). The fluorescence intensity of anti-PER and anti-TIM antibody staining was quantified from individual neurons using ImageJ software and averaged for each group of circadian pacemaker neurons. Y-axis indicates the relative expression levels (%) of PER and TIM proteins, calculated by normalizing to the peak value in each graph (set as 100). White/black bars, LD cycles; gray/black bars, DD cycles. s-LNv, PDF-expressing small ventral lateral neurons; LNd, dorsal LN. *P < 0.05, **P < 0.01 and ***P < 0.001 as determined by Student’s *t*-test. Error bars indicate SEM. (**b**) Representative confocal images of small LNv in wild-type (*w*^*1118*^) and *crtc* mutants (*crtc*^*25-3*^). 1CT/2CT, circadian time in the first/second DD cycle.

Molecular rhythms in fly head extracts largely reflect the circadian pace of peripheral clock tissues (i.e., photoreceptor neurons in the eye)^43^ and are not necessarily coupled to rhythmic behaviors in DD. Therefore, we assessed the circadian expression of PER and TIM proteins in behaviorally relevant pacemaker neurons by immunofluorescence assays. These clock neurons include dorsal LN (LNd) and PDF-expressing LNv (see Fig. 2a). In LD cycles, PER and TIM oscillations in the pacemaker neurons were relatively comparable between wild-type and *crtc* mutant flies (Fig. 4a, top). PER levels were significantly higher in LNd and large LNv of *crtc* mutants during the early L phase (Supplementary Fig. 9) but the phase difference in PER rhythms was subtle in small LNv. Similarly, there was no apparent phase-delay in TIM cycling yet the overall levels of TIM were modestly reduced in small LNv of *crtc* mutants. In contrast, ~8-hour phase-delays in PER and TIM rhythms were evident in the pacemaker neurons of *crtc* mutants during the transition from the first to the second DD cycle (Fig. 4a,b) as comparable to those in head extracts. These phase-delaying effects of *crtc* seem not due to the failure in the nuclear translocation of PER and TIM proteins (Fig. 4b). In addition, the strongest effects of *crtc* were observed in small LNv since the peak levels of the delayed TIM cycling substantially dampened in DD, consistent with long but poor rhythmicity in free-running behaviors. Taken together, these data suggest that *Drosophila* clocks require CRTC to timely induce clock gene expression in both central clock neurons and peripheral clock tissues. Nonetheless, the differential impact of *crtc* on the robustness of molecular rhythms (e.g., the amplitude of TIM cycling in head extracts versus small LNv) may arise from cell-type specific context of CRTC-dependent transcription.

### CRTC potentiates CLK-CYC–dependent *tim* transcription

On the basis of gene expression analyses in *crtc* mutants, we hypothesized that CRTC might facilitate a rate-limiting circadian transcription that coordinates molecular rhythms with 24-hour periodicity. However, the interlocked nature and feedback regulation of circadian transcription factors as well as the possibility of genetic compensation in mutant animals make it difficult to discriminate direct CRTC targets from indirect *crtc* effects on circadian transcription. To determine if the overall phase-delay in *crtc* mutants results from the attenuated transcription of general clocks (i.e., CLK-CYC–dependent clock gene expression) or a specific CRTC target, we tested *crtc* effects on individual clock reporter genes in clock-less *Drosophila* S2 cells. Of note, S2 cells endogenously express the *cyc* gene, so CLK overexpression alone is sufficient to support the transcriptional induction of CLK-CYC target reporter genes, such as *per*-luciferase (luc) or *tim*-luc^44^.

**Figure 5:**
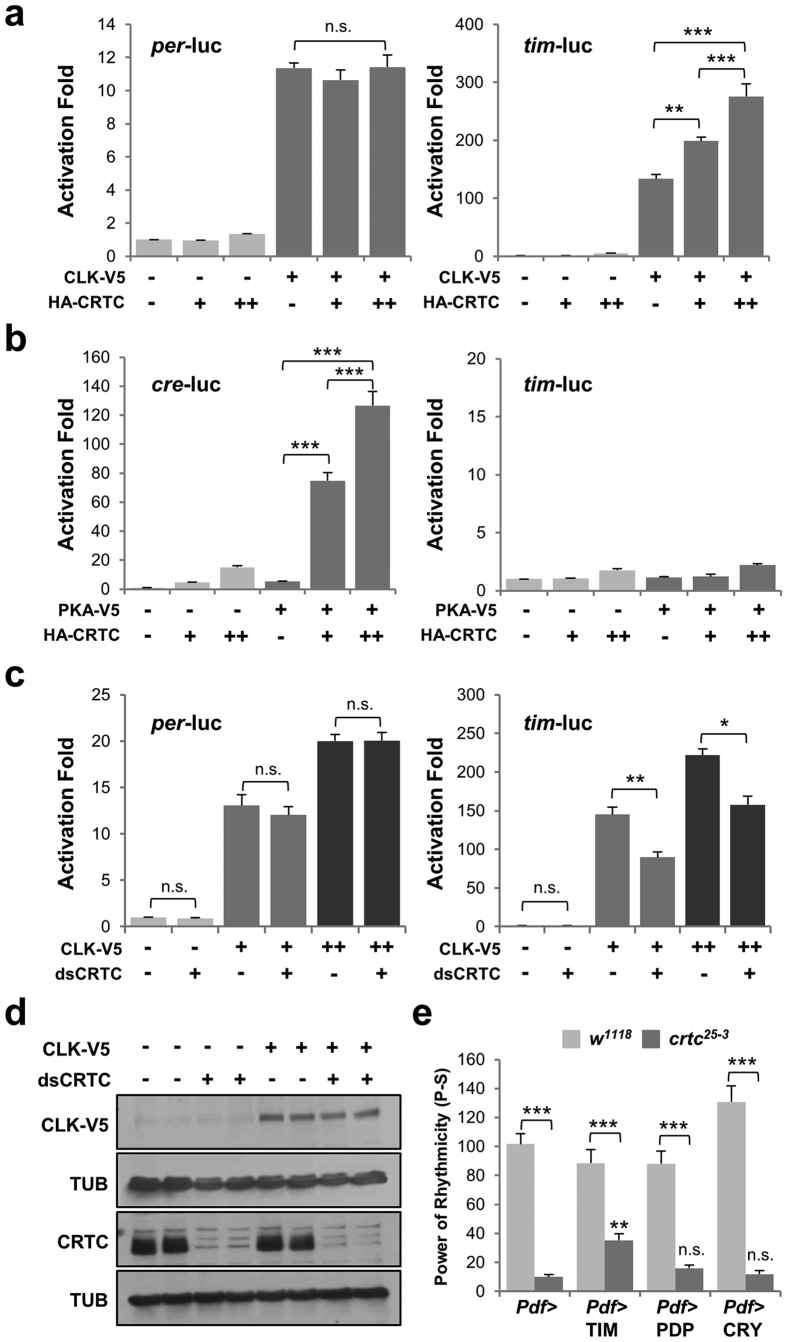
*timeless* is a primary clock target of *Drosophila* CRTC. (**a**) *Drosophila* S2 cells in 12-well plates were co-transfected with reporter plasmids (50 ng of *per*-luc or *tim*-luc; 50 ng of *renilla* luciferase) and expression vectors for V5-tagged CLK (0.2 ng) and HA-tagged CRTC (0, 50 or 250 ng). Dual luciferase reporter assays were performed 40 hours after transfection. Firefly luciferase activity was first normalized to that of *renilla* luciferase. Relative fold-activation was then calculated relative to baseline luciferase activity in the absence of any effectors. Data represent the average from three independent experiments. Error bars indicate SEM. n.s., not significant; **P < 0.01, ***P < 0.001 as determined by one-way ANOVA, Tukey post hoc test. (**b**) Reporter plasmids (50 ng of *cre*-luc or *tim*-luc; 50 ng of *renilla* luciferase) and expression vectors for V5-tagged PKA (5 ng) and HA-tagged CRTC (0, 50 or 250 ng) were cotransfected into S2 cells in 12-well plates. Data represent average +/− SEM (n = 3). ***P < 0.001 as determined by one-way ANOVA, Tukey post hoc test. (**c**) S2 cells in 12-well plates were pre-incubated with 10 μg of double-stranded RNAs against CRTC (dsCRTC) or EGFP (dsEGFP, control). Forty-eight hours after dsRNA treatment, cells were co-transfected with reporter plasmids (50 ng of *per*-luc or *tim*-luc; 50 ng of *renilla* luciferase) and expression vector for V5-tagged CLK (0.2 and 0.5 ng). Data represent average +/− SEM (n = 3). n.s., not significant, *P < 0.05, **P < 0.01 as determined by Student’s *t*-test. (**d**) dsRNA-treated S2 cells were transfected with 10 ng of V5-tagged CLK expression vector. Cell extracts were prepared 2 days after transfection, resolved by SDS-PAGE and immunoblotted for specific proteins under the same experimental conditions. Images were cropped from full-length blots shown in Supplementary Fig. 13. (**e**) TIM overexpression in PDF neurons rescues circadian behaviors in *crtc* mutants. Each clock gene was overexpressed in PDF neurons of wild-type or *crtc* mutants flies. Rhythmicity in DD locomotor behaviors was measured as in Fig. 1d. Data represent average +/− SEM (n = 16–57). n.s., not significant, **P < 0.01 to *Pdf*-Gal4 controls in *crtc* mutants as determined by one-way ANOVA, Tukey post hoc test.

In our S2 reporter assays, CRTC overexpression activated CLK-induced expression of *tim*-luc, but not *per*-luc, in a dose-dependent manner (Fig. 5a). Lack of *crtc* effects on *per*-luc was not due to the saturating activation of the reporter gene by CLK overexpression (Supplementary Fig. 8). To confirm that *crtc* effects indeed require CLK, we tested if CREB-dependent transcription could stimulate the expression of *tim*-luc in the absence of ectopic CLK. We first validated that CRTC and cAMP-dependent protein kinase (PKA) synergistically activated the expression of *cre*-luc, a reporter gene whose transcription is driven by tandem repeats of cAMP-responsive element (CRE), thus faithfully responding to CREB-dependent transcription in S2 cells (Fig. 5b, left). Under similar conditions, however, CRTC and PKA did not activate the baseline transcription of *tim*-luc, *Clk*-luc or *per*-luc (Fig. 5b, right; Supplementary Fig. 10), verifying that CLK-dependent *tim* transcription is a prerequisite for *crtc* effects. Moreover, RNAi-mediated CRTC depletion suppressed CLK-dependent activation of *tim*-luc but not *per*-luc (Fig. 5c). We confirmed that CRTC depletion did not affect ectopic CLK expression itself (Fig. 5d), further supporting that CRTC functions as a *tim* promoter-specific co-activator for CLK-dependent transcription.

These *in vitro* results are consistent with TIM dampening in the clock neurons of *crtc* mutants (Fig. 4a, bottom). Given that TIM post-translationally stabilizes PER via a protein-protein interaction^45^,^46^, it is likely that the lower amplitude of TIM rhythms could be limiting for robust PER cycling in the pacemaker neurons of *crtc* mutants. The *crtc* effects on the end-point measurements of CLK-activated *tim*-luc expression are also consistent with the phase-delays in *tim* mRNA cycling in head extracts of *crtc* mutants (i.e., delays in the full induction of *tim*) since lack of CRTC failed to potently induce *tim* transcription within a short window of its increasing phase. We thus reasoned that if *tim* transcription is rate-limiting in *crtc* mutants, TIM overexpression would rescue their circadian behaviors. To test this hypothesis, we overexpressed TIM or other clock proteins in PDF neurons of *crtc* mutants and examined their free-running locomotor behaviors in DD cycles. Indeed, TIM overexpression partially, but significantly, rescued long but weak behavioral rhythms in *crtc* mutants, whereas PDP1 or CRY (CRYPTOCHROME) overexpression did not show any comparable rescue particularly on the rhythmicity phenotype (Fig. 5e, Supplementary Table 7). Taken together, these data convincingly support our conclusion that CRTC is a novel co-activator for the CLK-activated *tim* transcription to sustain 24-hour rhythms in *Drosophila* circadian clocks.

## Discussion

CREB-dependent transcription has long been implicated in different aspects of circadian gene expression. In mammalian clocks, light exposure triggers intracellular signaling pathways that activate CREB-dependent *Per1* transcription, thereby adjusting the circadian phase of master circadian pacemaker neurons in the suprachiasmatic nucleus (SCN)^18^,^19^,^20^. The phase-resetting process involves the specific CREB coactivator CRTC1 and its negative regulator SIK1, constituting a negative feedback in the photic entrainment via a CREB pathway^21^,^24^. In this report, we demonstrated a novel role of *Drosophila* CRTC that serves to coordinate circadian gene expression with 24-hour locomotor rhythms even in the absence of light. CRTC may regulate several clock-relevant genes, including those clock output genes that might be involved in the rhythmic arborizations and PDF cycling of the circadian pacemaker neurons. However, we identified *tim* transcription as one of the primary targets of *Drosophila* CRTC to sustain circadian rhythms in the free-running conditions, thus defining its light-independent clock function.

CREB could employ another transcriptional coactivator CBP (CREB-binding protein) to activate CRE-dependent transcription^47^,^48^. In fact, CBP is a rather general coactivator recruited to gene promoters by other DNA-binding transcription factors^49^. We have previously shown that *Drosophila* CBP associates with CLK, titrating its transcriptional activity^50^. Mammalian CBP and the closely related coactivator p300 also form a complex with CLOCK-BMAL1, a homolog of the *Drosophila* CLK-CYC heterodimer, to stimulate their transcriptional activity^51^,^52^. One possible explanation for CRTC-activated *tim* transcription is that *Drosophila* CRTC may analogously target the CLK-CYC heterodimer to stimulate CLK-CYC–dependent *tim* transcription. However, this model does not explain *tim*-specific *crtc* effects among other CLK-CYC–induced clock genes. Moreover, CRTC associates with the bZIP domain in CREB protein, whereas CBP/p300 binds CREB through the phosphorylated KID domain^53^,^54^, indicating that they might not necessarily target the same transcription factors apart from CREB. Finally, we could not detect a protein complex of CLK and CRTC in *Drosophila* S2 cells (Supplementary Fig. 11). Thus, it is likely that CRTC and CBP/p300 play unique roles in circadian transcription through their interactions with different DNA-binding transcription factors.

**Figure 6:**
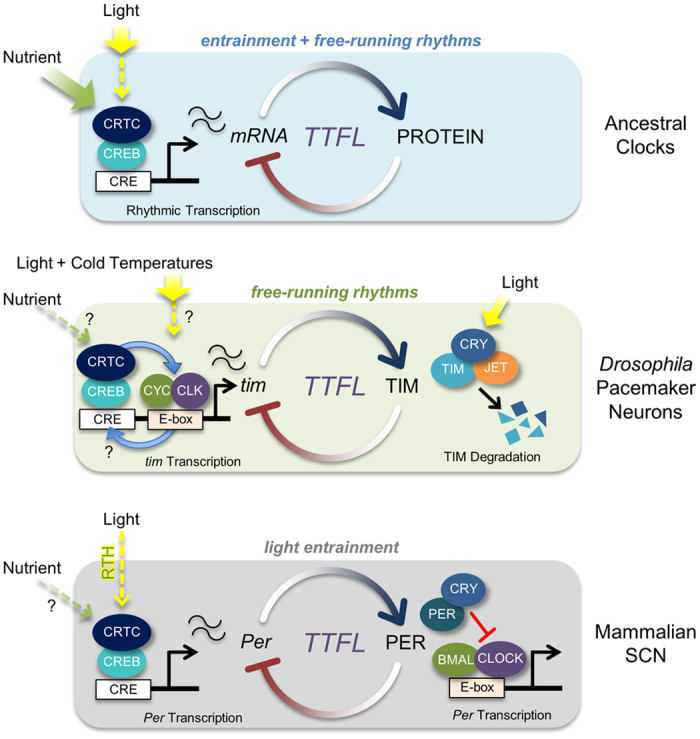
A model for the evolution of CRTC-dependent clocks. In ancestral organisms, environmental time cues such as light or the availability of nutrients might have been directly accessible to circadian clock cells. Timing information could have converged on the regulation of CRTC through various intracellular signaling pathways to modulate CREB-dependent transcription in the earliest transcription-translation feedback loop (TTFL), thereby entraining and sustaining molecular clocks. In poikilothermic *Drosophila*, environmental changes in light could be cell-autonomously sensed by circadian pacemaker neurons, whereas metabolic cues are instead provided systemically. Accordingly, post-translational regulation of TIM stability has evolved as the primary strategy for clock entrainment by light since the blue-light photoreceptor CRY and the F box protein JETLAG (JET) trigger light-dependent TIM degradation. CREB-dependent transcriptional regulation of the *tim* promoter, on the other hand, has been conserved from the original TTFL. Given that *Drosophila tim* is essential for sustaining molecular rhythms, a transcriptional co-activator function of CRTC in CLK/CYC-induced *tim* transcription contributes to free-running behaviors in DD. In homeothermic mammals, light cues are indirectly transmitted to master pacemaker neurons in the suprachiasmatic nucleus (SCN) by the synaptic input from the retinohypothalamic tract (RHT). Consequently, the mammalian *tim* homolog became dispensable for clock function whereas the light-sensing activity of CRY homologs has been replaced by their transcriptional repressor function. Instead, mammalian *Per* took over a role in light entrainment by retaining the CREB/CRTC-dependent transcriptional regulation from the primitive TTFL.

If CRTC augments CLK-CYC–dependent *tim* transcription indirectly, then why do *crtc* effects require CLK? A recent study suggested that mammalian CLOCK-BMAL1 may regulate the rhythmic access of other DNA-binding transcription factors to their target promoters in the context of chromatin, acting as a pioneer-like transcription factor^55^. Given the structural and functional homology between *Drosophila* CLK-CYC and mammalian CLOCK-BMAL1, the presence of CLK-CYC in the *tim* promoter might allow the recruitment of additional transcription factors (e.g., CREB) and their co-activators including CRTC for maximal *tim* transcription (Fig. 6, middle). The transcriptional context of *tim* promoter might thus define its sensitivity to *crtc* effects among other clock promoters. In addition, the differential assembly of transcription factors on the *tim* promoter could explain tissue-specific effects of *crtc* on TIM oscillations (i.e., circadian pacemaker neurons versus peripheral clock tissues). Interestingly, chromatin immunoprecipitation with V5-tagged CLK protein revealed that CLK-CYC heterodimers associate with both *tim* and *Sik2* gene promoters in fly heads^56^. In LD cycles, however, their rhythmic binding to the *Sik2* promoter is phase-delayed by ~4.5 hours compared with that to the *tim* promoter. These modes of transcriptional regulation may gate *crtc* effects on *tim* transcription in a clock-dependent manner, particularly in the increasing phase of *tim* transcription.

Transcription from CREB-responsive reporter genes shows daily oscillations, both in *Drosophila* and mammals^19^,^57^,^58^, implicating this transcriptional strategy in the evolution of molecular clocks. In fact, cAMP signaling and CRE-dependent transcription constitute the integral components of core molecular clocks, serving to regulate daily rhythmic transcription of circadian clock genes^57^,^58^,^59^,^60^,^61^. For instance, reciprocal regulation of *dCREB2* and *per* at the transcription level has been reported to sustain free-running circadian rhythms in *Drosophila*^57^. During fasting in mammals, a transcriptional program for hepatic gluconeogenesis is induced by CREB phosphorylation and CRTC2 dephosphorylation. Fasting-activated CREB-CRTC2 then stimulates *Bmal1* expression^61^, whereas CLOCK-BMAL1–induced CRY rhythmically gates CREB activity in this process by modulating G protein-coupled receptor activity and inhibiting cAMP-induced CREB phosphorylation^62^. This molecular feedback circuit thus mutually links mammalian clocks and energy metabolism in terms of CREB-dependent transcription.

On the basis of these observations, we propose a model for the evolution of CRTC-dependent clocks to explain the distinctive circadian roles of CRTC homologs (Fig. 6). CRTC is a transcriptional effector that integrates various cellular signals^33^. We reasoned that ancestral clocks may have employed CREB-CRTC–mediated transcription to sense extracellular time cues cell-autonomously and integrate this timing information directly into the earliest transcription-translation feedback loop (TTFL). This strategy would have generated simple but efficient molecular clocks to tune free-running molecular rhythms in direct response to environmental zeitgebers, such as light and the availability of nutrients. A circadian role of CRTC then has differentially evolved along with a selective set of clock targets. In poikilothermic *Drosophila*, light is accessible directly to circadian pacemaker neurons in the adult fly brain. Therefore, TIM degradation by the blue-light photoreceptor CRY^63^,^64^ plays a major role in the light entrainment of *Drosophila* clocks although the photic induction of CLK/CYC-dependent *tim* transcription has been reported specifically at lower temperatures^65^. Accordingly, *Drosophila* CRTC retained a constitutive co-activator function from the ancestral TTFL to support CLK/CYC-activated *tim* transcription and sustain free-running circadian behaviors. In homeothermic mammals, light input to the SCN is indirectly mediated by neurotransmitter release from presynaptic termini of the retinohypothalamic tract (RHT)^66^. Intracellular signaling relays in the SCN converge on the dephosphorylation and nuclear translocation of CRTC1 to activate CRE-dependent transcription^21^,^24^. Under these circumstances, a circadian role of light-sensitive TIM might have degenerated, while *per* took over a role in the light-entrainment pathway by retaining CREB-CRTC1–dependent transcriptional regulation from the primitive TTFL. Consequently, mammalian clocks have lost a homolog of the *Drosophila*-like *cry* gene family, but instead evolved CRY homologs of the vertebrate-like *cry* gene family with transcriptional repressor activities in CLOCK-BMAL1–dependent transcription^67^.

Regulation of metabolism and stress responses by neuronal CREB-CRTC-SIK pathways has been well documented in *Drosophila*^25^,^34^,^68^. Given our demonstration of a circadian role of CRTC in the pacemaker neurons, it is possible that CRTC might sense metabolic cues in the context of circadian neural circuits to entrain molecular clocks cell-autonomously. Alternatively, but not exclusively, CRTC could participate in the regulation of clock-relevant metabolism as clock outputs from pacemaker neurons^69^,^70^. These hypotheses remain to be validated in future studies.

## Materials and Methods

### Fly Strains

All flies were reared on standard cornmeal-yeast-agar medium at 25 °C under 12 hour:12 hour LD cycles. *crtc*^*25-3*^ and UAS-CRTC (gifts from M. Montminy), as well as UAS-SIK (a gift from J. Chung), *Pdf*-GeneSwitch-Gal4 (a gift from M. Ceriani), *Pdf*-Gal4, and *tim*-Gal4 have been described previously^25^,^32^,^34^,^38^,^71^. *Df*(3L)ED4710*, Df*(3L)BSC415, UAS-*crtc* RNAi (stock number 28886), and UAS-DCR2 were obtained from the Bloomington *Drosophila* Stock Center.

### Behavioral Analyses

Individual male flies were placed into glass vials containing 5% sucrose and 2% agar, entrained by three LD cycles at 25 °C and then transferred to constant darkness (DD). Locomotor activities were recorded using the *Drosophila* Activity Monitor (DAM) system (TriKinetics). Behavioral data were collected from the first to the 6th DD cycle and analyzed to determine the free-running period length and power of rhythmicity in individual flies using the ClockLab analysis software (Actimetrics). The confidence interval of the chi-squared periodogram was set to 0.05 with the testing range of period lengths from 15-hour to 35-hour. The power of rhythmicity (P-S) was calculated by subtracting a Significance value (the minimum measurement considered rhythmic at a given period length with the confidence interval) from a Power value (the observed rhythmicity measurement at the given period length). All the genotypes were tested in multiple behavioral runs. Per each genotype, circadian periods were averaged from rhythmic flies only (which are operationally defined if flies have the power values of rhythmicity greater than 10) whereas the power of rhythmicity values were averaged either from all the flies tested or from rhythmic flies as shown in Supplementary Tables. The averaged locomotor activity profiles were analyzed using Microsoft Excel. To quantify the anticipatory morning activities in LD cycles, we first calculated the normalized total activities from individual flies and then applied them to the equation for the morning index = [(total activity 3 h prior to lights-on)/(total activity 6 h prior to lights-on) − (0.5)].

### Immunoblotting

Fifty fly heads were homogenized in lysis buffer consisting of 25 mM Tris-Cl pH 7.5, 300 mM NaCl, 10% glycerol, 1 mM PMSF, 1 mM DTT, 0.5% NP-40, and protease/phosphatase inhibitor cocktail. Soluble extracts were clarified by centrifugation, resolved by sodium dodecyl sulfate-polyacrylamide gel electrophoresis (SDS-PAGE), and transferred to nitrocellulose membranes (Amersham) for immunoblotting. Membranes were probed with rabbit anti-CRTC (a gift from J. Chung), guinea pig anti-TIM^50^, rabbit anti-PER (a gift from E.Y. Kim), mouse anti-TUB (Developmental Studies Hybridoma Bank) and anti-V5 (Invitrogen) primary antibodies, and detected using appropriate horseradish peroxidase-conjugated secondary antibodies and enhanced chemiluminescence (ECL) reagents (Pierce).

### Immunofluorescence Assay

Male flies were entrained in three LD cycles and then transferred to constant darkness. Brains were dissected at six different time-points in LD cycles or from the first to the second DD cycle. Whole-mount immunostaining was performed as described previously^72^,^73^. Rabbit anti-PER (a gift from E. Y. Kim), guinea pig anti-TIM^50^ and mouse anti-PDF (Developmental Studies Hybridoma Bank) were used as primary antibodies, and Alexa Fluor 488-conjugated anti-rabbit IgG, Alexa Fluor 594-conjugated anti-guinea pig IgG, and Alexa Fluor 647-conjugated anti-mouse IgG (Jackson Immuno Research Laboratories, Inc.) were used as secondary antibodies. Brain images were obtained by confocal laser-scanning microscopy (Olympus FV1000). For quantitative analyses of PER and TIM levels in the circadian pacemaker neurons, the fluorescence intensity from each group of clock cells was quantified using ImageJ software, as described previously^72^. The quantification of the axonal arborizations from small LNv was performed as described previously^36^. To quantify PDF levels at the axonal termini of small LNv, the intensities of anti-PDF staining were quantified using ImageJ software. Those above a threshold levels were integrated from each z-stacked confocal images and averaged from 16–20 hemispheres per each genotype at ZT0 or ZT12.

### Quantitative Real-time PCR

Flies were entrained in three LD cycles and then transferred to constant darkness. For transcription analyses of clock genes, flies were harvested at six different time-points in LD cycles or during the transition from the first to the second DD cycle. Total RNA was extracted from 50 fly heads using TRIzol reagent (Ambion), according to the manufacturer’s instructions. Purified RNA was digested with RQ1 DNase to remove genomic DNAs, extracted with a mixture of phenol:chloroform:isoamyl alcohol (25:24:1), and then reverse-transcribed with M-MLV reverse transcriptase using oligo dT primers, according to the manufacturer’s instructions (Promega). Quantitative polymerase chain reaction (PCR) was performed on a CFX96 system (Bio-Rad) using TOPreal qPCR 2X PreMIX (Enzynomics) and the following specific primer pairs: *rp49* (control), 5′-GAA GAA GCG CAC CAA GCA CT-3′ (forward) and 5′-TTG AAT CCG GTG GGC AGC AT-3′ (reverse); *tim*, 5′-GAC TTG CCA AAT CCC TCA TC-3′ (forward) and 5′-GAA GCA CTG CAA CTC GAT CA-3′ (reverse); *per*, 5′-GAC CGA ATC CCT GCT CAA TA-3′ (forward) and 5′-GTG TCA TTG GCG GAC TTC TT-3′ (reverse); *Clk*, 5′-TAC TGC GTG AGG ATA TCG-3′ (forward) and 5′-GTT GTT GTT CTG GTT GC-3′ (reverse); *Pdp1e*, 5′-GTC GCC CTC CTC CTT GTA TT-3′ (forward) and 5′-CCA ATG AGC ATC ACA ACC AT-3′ (reverse); *cry*, 5′-CCA CCG CTG ACC TAC CAA A-3′ (forward) and 5′-GGT GGA AGC CCA ATA ATT TGC-3′ (reverse); and *pdfr*, 5′-CAG CTC GTT AGC ATT GTC CA-3′ (forward) and 5′-ACG TTT AAG TTG GCC ACA GG-3′ (reverse).

### S2 Cell Culture, Plasmids, Transfection, and Luciferase Assay

*Drosophila* S2 cells were cultured in Shields and Sang M3 insect medium (Sigma) supplemented with 10% fetal bovine serum and 1% penicillin-streptomycin (Invitrogen). Transient transfection was performed using Effectene according to the manufacturer’s instructions (Qiagen). *crtc* cDNA (a gift from J. Chung) was subcloned into a modified pAc vector for expression of HA-tagged CRTC. pAc-CLK-V5, pAc-PKA-V5, *per*-luc*, tim*-luc, *Clk*-luc, *cre*-luc and *renilla*-luc vectors have been described previously^10^,^44^,^50^. For S2 RNAi experiments, double-stranded RNAs (dsRNAs) were *in vitro*-transcribed using a MEGAscript RNAi kit (Ambion). S2 cells were treated with dsRNA against EGFP (control) or CRTC for 2 days before transfection. Luciferase assays were performed 44 hours after transfection using the Dual-Luciferase Reporter Assay System according to the manufacturer’s instructions (Promega). Luminescence was measured using a TriStar2 plate reader (Berthold Technologies).

## Additional Information

**How to cite this article**: Kim, M. *et al*. CRTC Potentiates Light-independent *timeless* Transcription to Sustain Circadian Rhythms in *Drosophila*. *Sci. Rep.* **6**, 32113; doi: 10.1038/srep32113 (2016).

## Supplementary Material

Supplementary Information
