# Supplementary material for: CRTC Potentiates Light-independent timeless Transcription to Sustain Circadian Rhythms in Drosophila
Source: Sci Rep. 2016 Aug 31;6:32113. doi: 10.1038/srep32113 (PMC5005998; doi:10.1038/srep32113)
Supplement: Supplementary Information [file srep32113-s1.pdf]

CRTC Potentiates Light-independent *timeless* Transcription to Sustain Circadian Rhythms in *Drosophila*

Minkyung Kim[1], Hoyeon Lee[2], Jin-Hoe Hur[3], Joonho Choe[1,*] and Chunghun Lim[2,*]

[1]Department of Biological Sciences, Korea Advanced Institute of Science and Technology, Daejeon 34141, Republic of Korea
[2]School of Life Sciences, Ulsan National Institute of Science and Technology (UNIST), Ulsan 44919, Republic of Korea
[3]UNIST-Olympus Biomed Imaging Center (UOBC), UNIST, Ulsan 44919, Republic of Korea

*Correspondence: jchoe@kaist.ac.kr or clim@unist.ac.kr

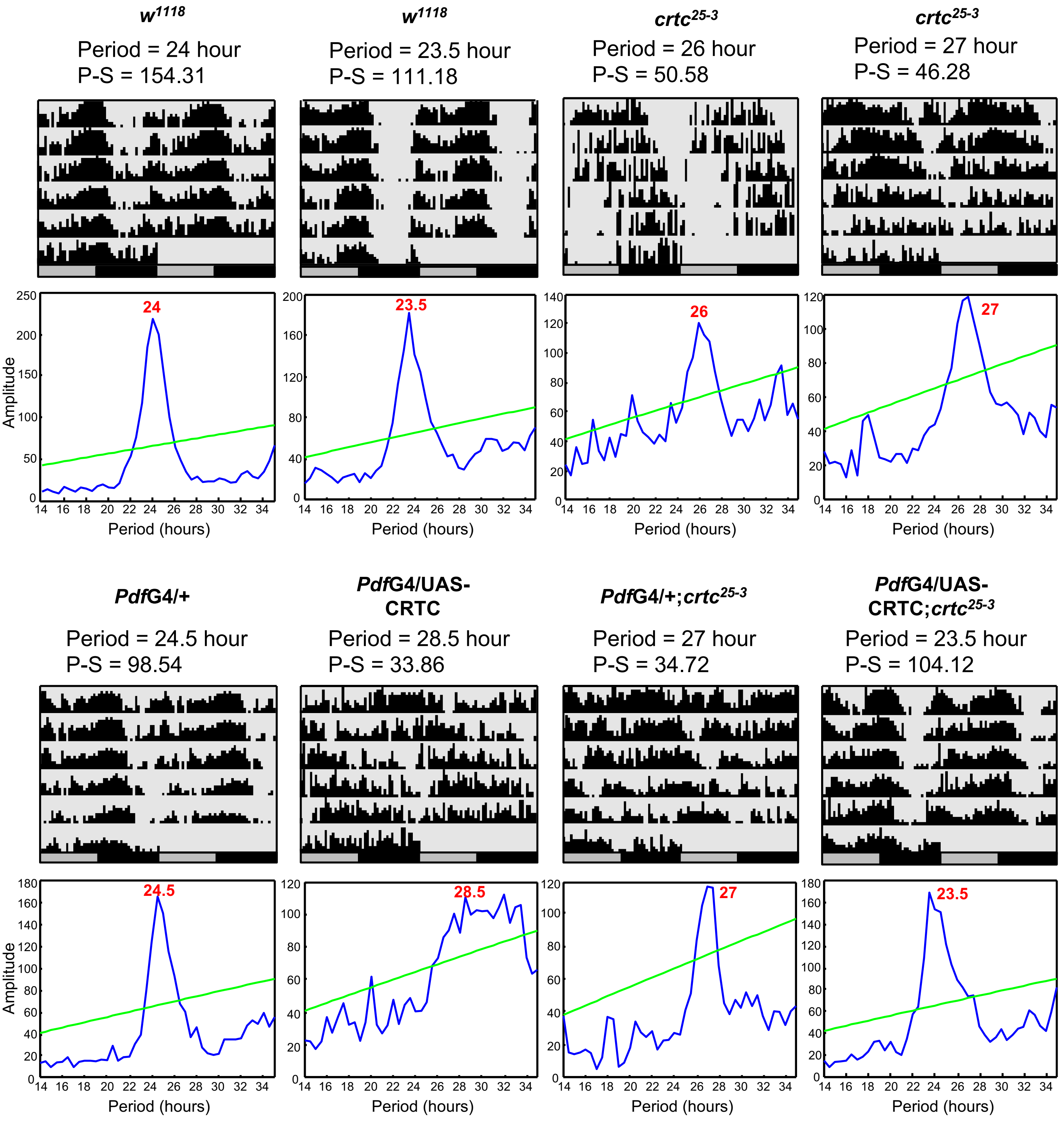

Supplementary Figure 1. Long-period rhythms are evident in free-running locomotor behaviors of individual flies with *crtc* mutant backgrounds

Locomotor behaviors were monitored for six DD cycles after LD entrainment. Representative actograms from individual flies were double-plotted with their genotypes at the top while the chi-square periodograms were shown with the significant lines (green) at the bottom. Circadian period and rhythmicity (P-S) in DD locomotor behaviors were measured similarly as in Fig. 1. Gray/black bars, DD cycles.

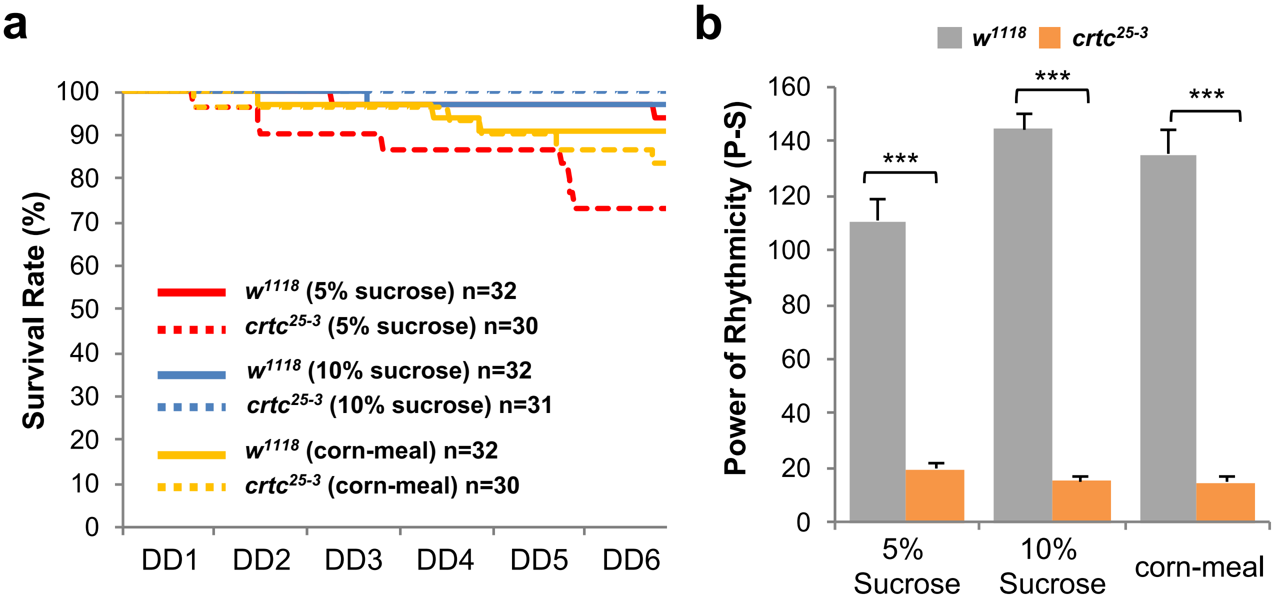

Supplementary Figure 2. Enriched food rescues lower survival rate in *crtc* mutants but not their poor rhythmicity in the free-running locomotor behaviors
(**a**) Survival rates in control (w^{1118}, solid lines) and *crtc* mutants ($crtc^{25\text{-}3}$, dotted lines) were measured during three LD cycles followed by six DD cycles. Where indicated, 5% sucrose (red), 10% sucrose (blue) or corn-meal food (yellow) were fed during the behavioral tests to determine circadian periods and rhythmicity. (**b**) Rhythmicity in DD locomotor behaviors was measured similarly as in Fig. 1d. ***$P<0.001$ as determined by Student's *t*-test. Error bars indicate SEM.

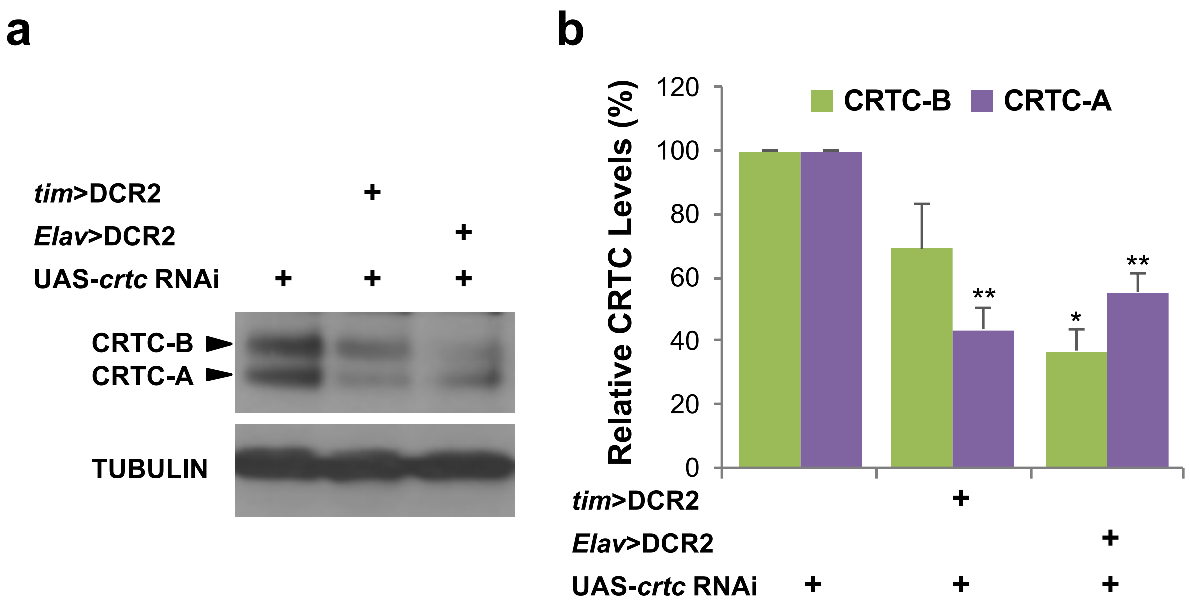

Supplementary Figure 3. *crtc* RNA interference (RNAi) transgene effectively reduce endogenous levels of CRTC proteins in fly heads

(**a**) A *crtc* RNAi transgene was overexpressed by clock neuron-specific *tim*-Gal4 or pan-neuronal *Elav*-Gal4 together with RNAi-enhancing *Dicer2* (*tim*>DCR2 or *Elav*>DCR2). Fly head extracts were prepared and immunoblotted with anti-CRTC and anti-TUBULIN (loading control) antibodies. Note that the anti-CRTC antibody detected two isoforms of CRTC proteins in control flies. Representative immunoblots for each antibody were shown. (**b**) Protein band intensities for each CRTC protein were measured using ImageJ software and normalized to those for tubulin. The relative levels of CRTC proteins in each genotype were calculated by normalizing to the values in control flies (UAS-*crtc* RNAi) which were set as 100%. Data represent the average of three independent experiments. **P*<0.05, ***P*<0.01 to UAS-*crtc* RNAi controls as determined by one-way ANOVA, Tukey post hoc test. Error bars indicate SEM.

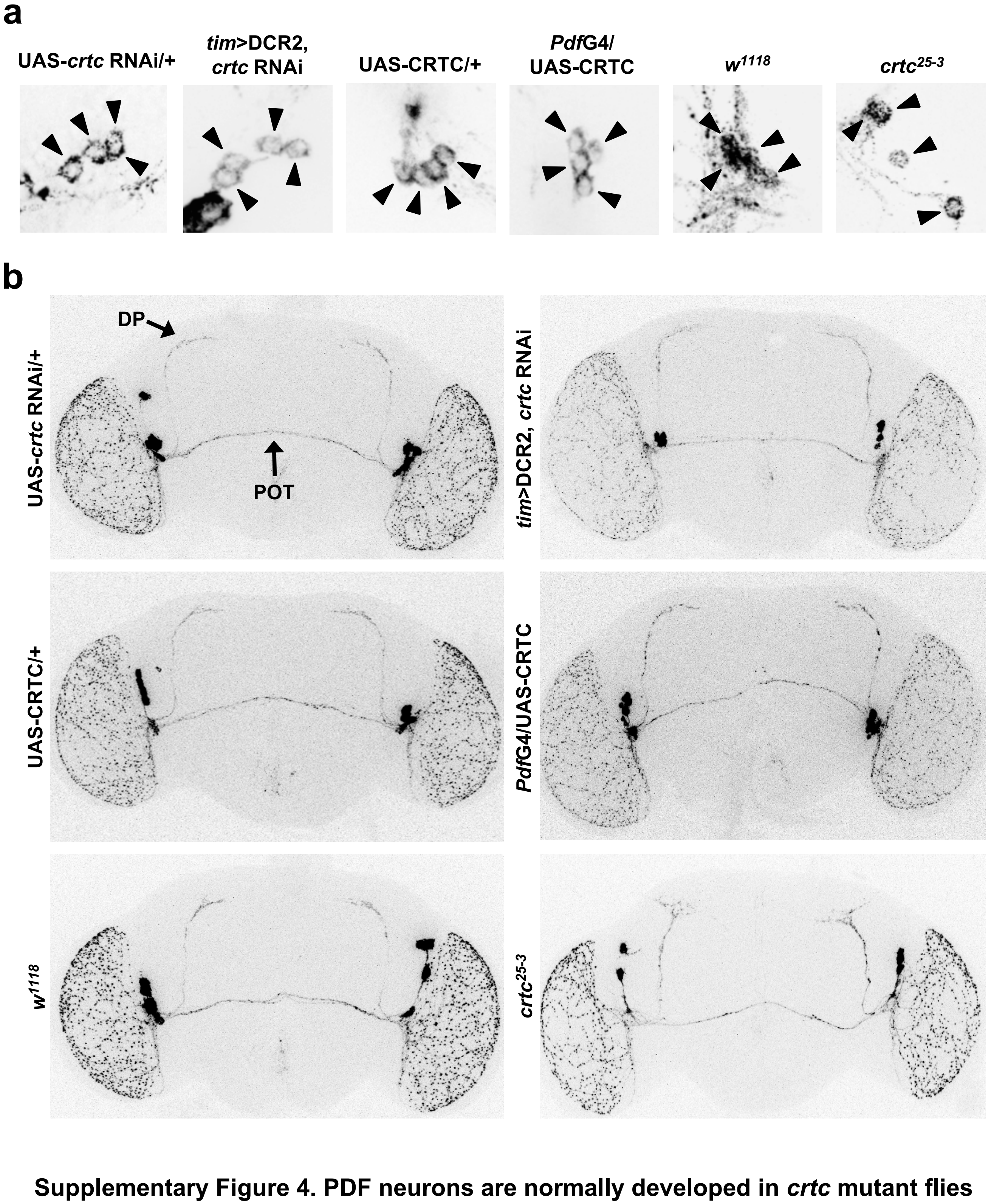

Supplementary Figure 4. PDF neurons are normally developed in *crtc* mutant flies
Adult fly brains were dissected at ZT3 in LD cycles and immunostained with anti-PDF antibody. Representative confocal images per each genotype were shown. (**a**) Cell bodies from small LNv were indicated by arrow heads. (**b**) Axonal projections from PDF-expressing neurons. POT, posterior optic tract from large LNv; DP, dorsal projections from small LNv

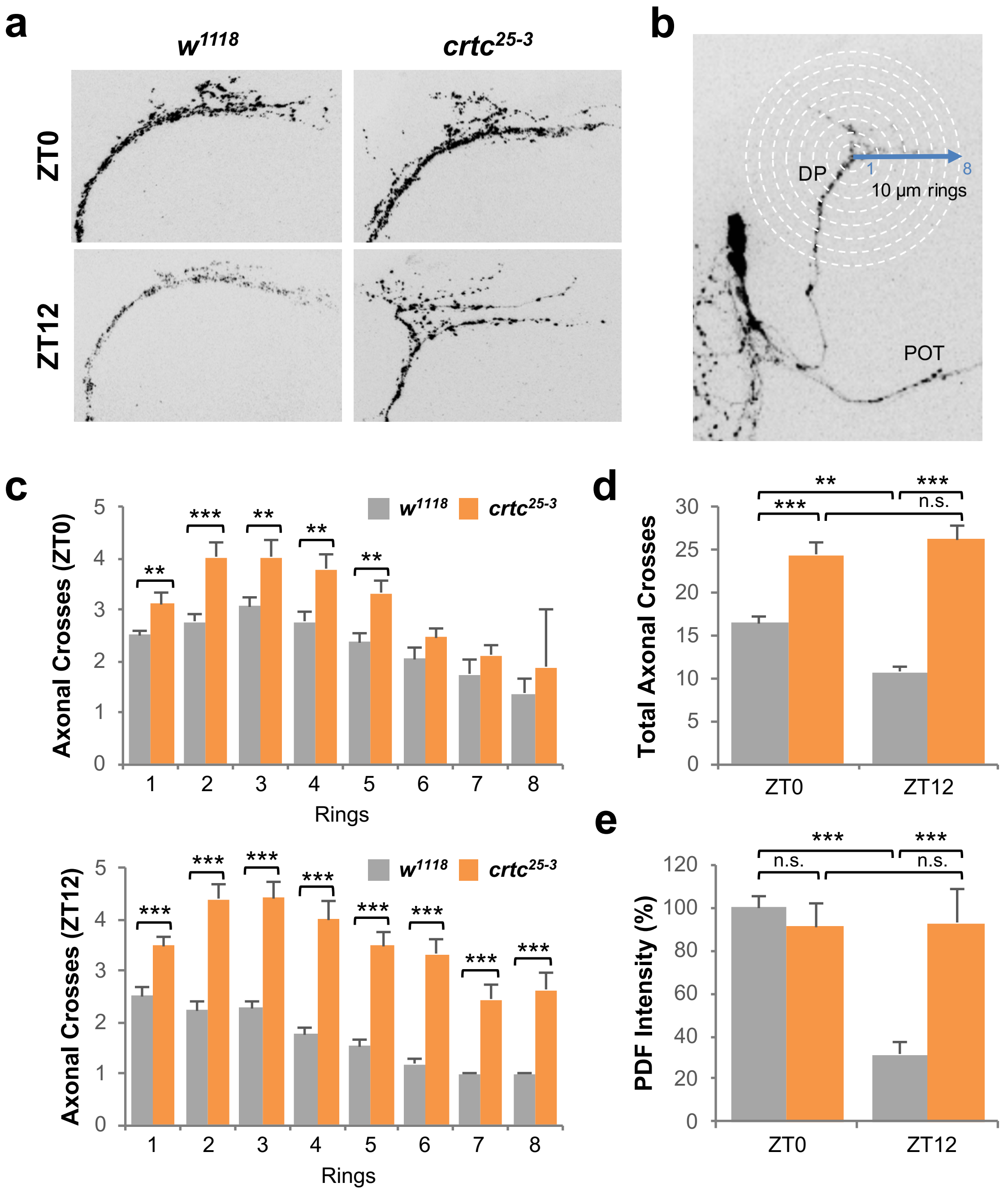

Supplementary Figure 5. The axonal termini of small LNv in *crtc* mutants constitutively display more arborizations and stronger PDF intensities

Adult fly brains were dissected at ZT0 and ZT12 in LD cycles and immunostained with anti-PDF antibody. (**a**) Representative confocal images of the dorsal projections from small LNv. (**b**) A diagram for the quantification of axonal arborizations by Sholl analysis[37]. POT, posterior optic tract from large LNv; DP, dorsal projections from small LNv. (**c**) Axonal crosses at each concentric ring were counted and averaged from 28-30 hemispheres per each genotype at ZT0 (top) or ZT12 (bottom). $^{**}P<0.01$, $^{***}P<0.001$ as determined by Student's *t*-test. Error bars indicate SEM. (**d**) Total axonal crosses per each hemisphere were calculated and averaged similarly as in (**c**). $^{**}P<0.01$, $^{***}P<0.001$ as determined by one-way ANOVA, Tukey post hoc test. Error bars indicate SEM. (**d**) The intensities of anti-PDF staining at the termini of DP above a threshold level were quantified using ImageJ software and averaged from 16-20 hemispheres per each genotype at ZT0 or ZT12. n.s., not significant; $^{***}P<0.001$ as determined by one-way ANOVA, Tukey post hoc test. Error bars indicate SEM.

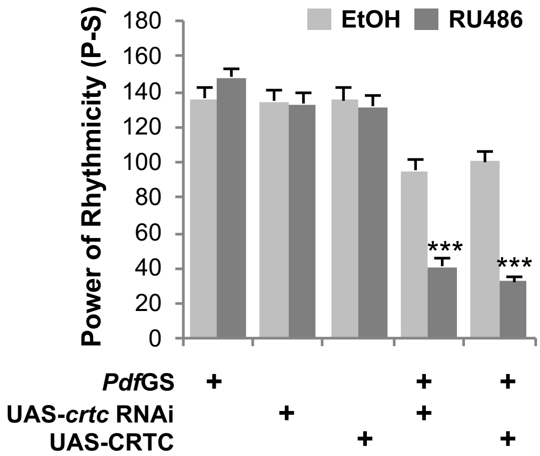

Supplementary Figure 6. *crtc* effects on circadian behaviors are adult-specific
Conditional depletion or overexpression of CRTC in PDF neurons of adult flies was sufficient to affect circadian behaviors in DD. Transgenic flies were fed on food containing 125 μM mifepristone (RU486) or 1% ethanol (vesicle control) during the period of behavioral tests. The RU486-activated *Pdf*-GeneSwitch (GS)-Gal4 driver transiently drove the overexpression of *crtc* RNAi or cDNA in PDF neurons. Rhythmicity in DD locomotor behaviors was measured similarly as in Fig. 1d. Light-gray bars, ethanol-fed flies; dark-gray bars, RU486-fed flies. ****P*<0.001 compared to ethanol-fed controls for Gal4/UAS trans-heterozygotes or all RU486-fed controls heterozygous for Gal4 or UAS transgene as determined by one-way ANOVA, Tukey post hoc test. Error bars indicate SEM.

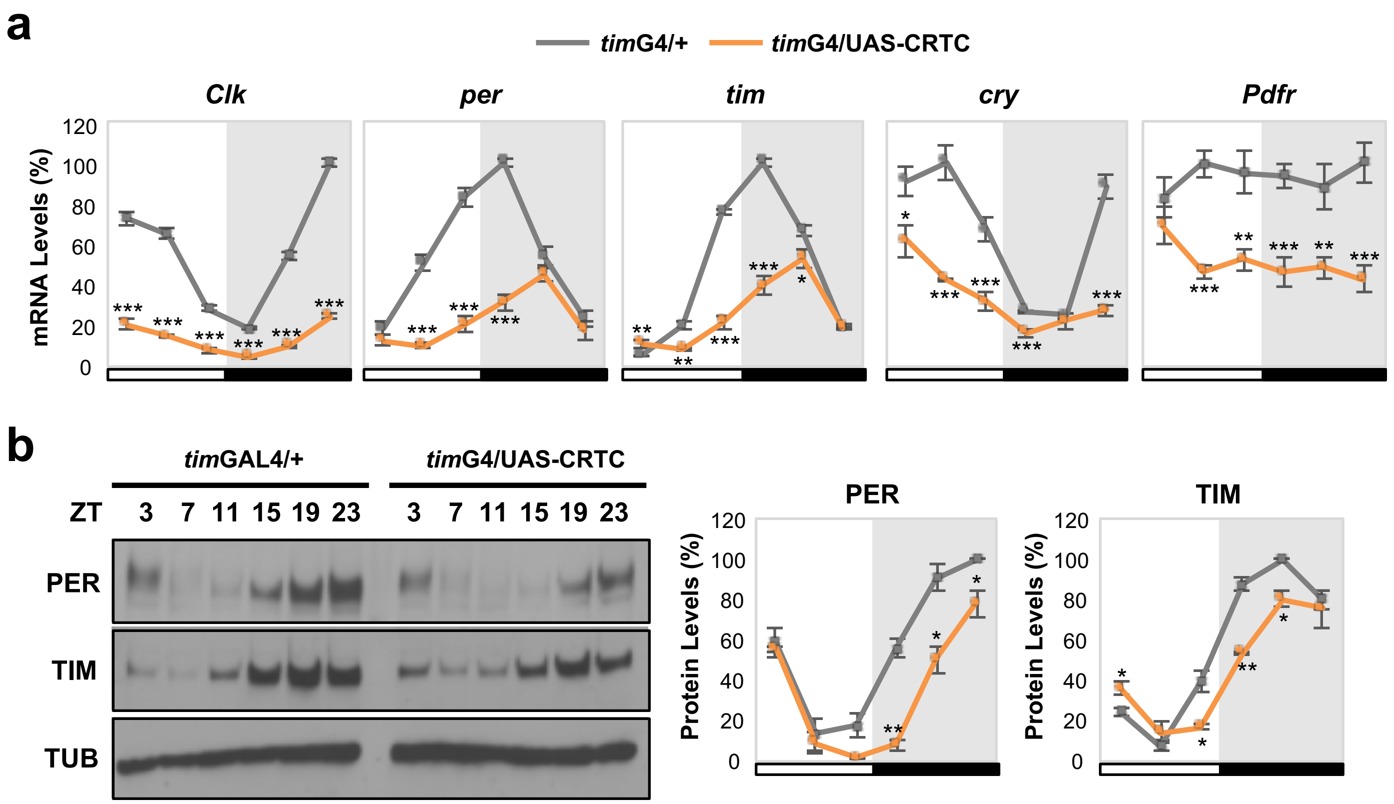

Supplementary Figure 7. CRTC overexpression in *tim*-expressing clock neurons dampens the amplitude of clock gene expression

(**a**) Circadian expression of clock-relevant mRNAs in adult heads of control (*tim*G4/+, gray lines) and CRTC-overexpressing flies (*tim*G4/UAS-CRTC, orange lines). Flies were collected at six different time-points in LD cycles and total RNAs were purified from their head extracts. Relative levels of *Clk*, *per*, *tim*, *cry*, and *Pdfr* (*Pdf* receptor) mRNAs were quantified by real-time RT-PCR. X-axis indicates zeitgeber time (ZT) in LD whereas Y-axis indicates relative expression levels (%) at each time-point, calculated by normalizing to the peak value (set as 100). (**b**) Circadian expression of PER and TIM proteins in adult fly heads. Head extracts were prepared from flies harvested during LD cycle and immunoblotted with anti-PER, anti-TIM and anti-TUBULIN (TUB, loading control) antibodies. Representative results of immunoblotting were shown on the left. Protein band intensities in each lane were quantified using ImageJ software and normalized to that of TUB protein. Y-axis indicates the relative expression levels (%) of PER and TIM proteins, calculated by normalizing to the peak value (set as 100). White/black bars, LD cycles. Data represent the average of three independent experiments. *P<0.05, **P<0.01 and ***P<0.001 as determined by Student's *t*-test. Error bars indicate SEM.

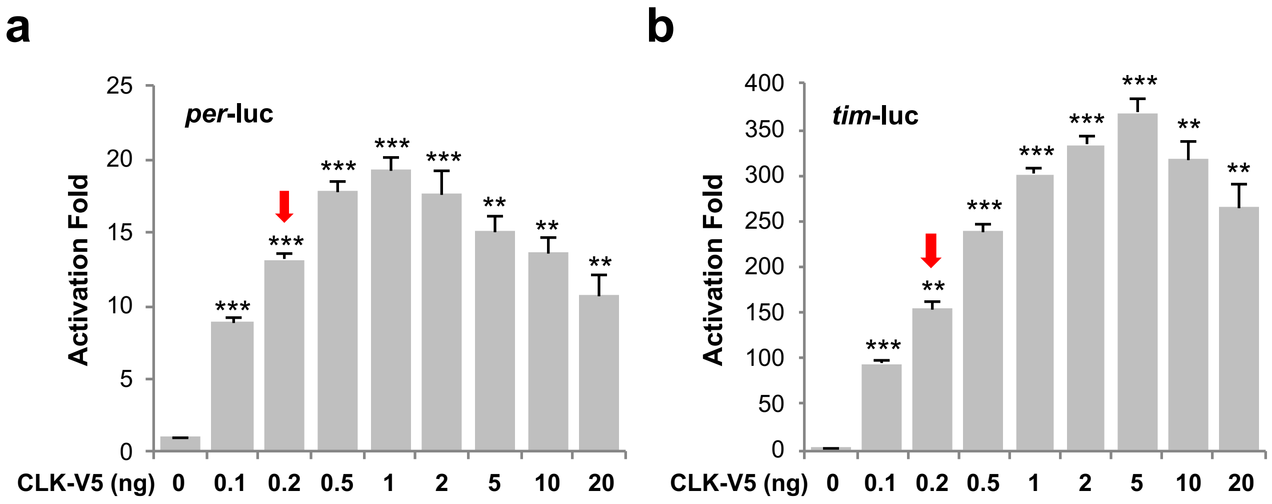

Supplementary Figure 8. Dose-response curves in CLK-activated transcription of reporter genes containing *per* and *tim* promoters

Drosophila S2 cells in 12-well plates were co-transfected with reporter plasmids (50 ng of *per*-luc (**a**) or *tim*-luc (**b**); 50 ng of renilla luciferase) and the increasing amounts of expression vector for V5-tagged CLK (0-20 ng). Dual luciferase reporter assays were performed 40 hours after transfection. Firefly luciferase activity was first normalized to that of renilla luciferase. Relative fold-activation was then calculated relative to baseline luciferase activity in the absence of CLK-V5. Given the linear range of CLK activation in both reporters, we used 0.2 ng of the CLK-V5 expression vector (indicated by red arrows) for the subsequent reporter assays in Fig. 5. Data represent the average from four independent experiments. **$P<0.01$, ***$P<0.001$ to the baseline luciferase activity in the absence of CLK-V5 as determined by Student's *t*-test. Error bars indicate SEM.

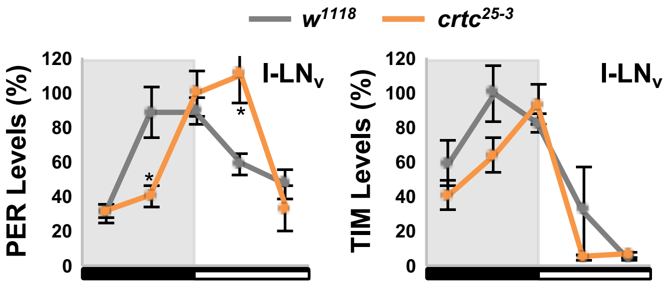

Supplementary Figure 9. *crtc* mutation impacts on PER and TIM oscillations in circadian pacemaker neurons

Circadian expression of PER and TIM proteins in large LNv of wild-type (gray lines) and *crtc* mutants (orange lines). Adult fly brains were dissected at different time-points in LD cycles. Whole-mount immunostaining was performed using anti-PER, anti-TIM, and anti-PDF antibodies. Confocal brain images were obtained from 12–13 hemispheres at each time-point (X-axis). The fluorescence intensity of anti-PER and anti-TIM antibody staining was quantified from individual neurons using ImageJ software and averaged for each group of circadian pacemaker neurons. Y-axis indicates the relative expression levels (%) of PER and TIM proteins, calculated by normalizing to the peak value in each graph (set as 100). White/black bars, LD cycles. l-LNv, PDF-expressing large ventral lateral neurons. *$P<0.05$ as determined by Student's *t*-test. Error bars indicate SEM.

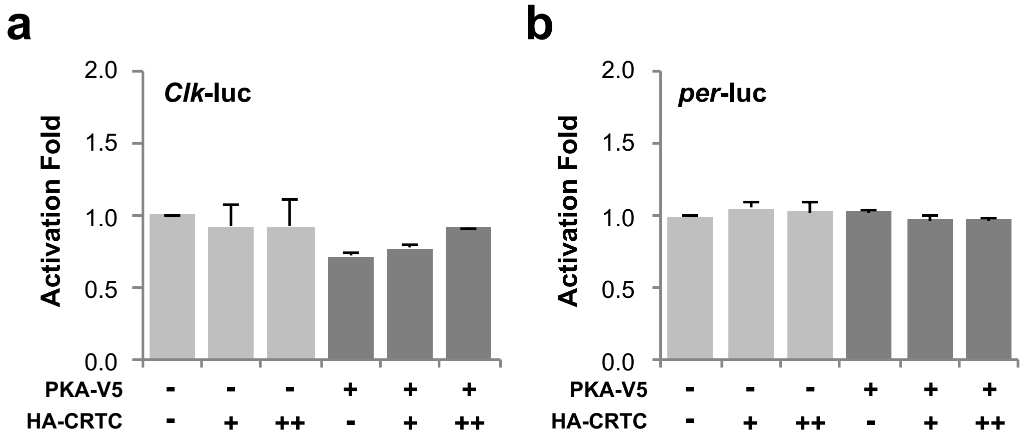

Supplementary Figure 10. Overexpression of CRTC and PKA does not affect the baseline transcription of *Clk*-luc or *per*-luc Reporter
S2 cells in 12-well plates were co-transfected with reporter plasmids (50 ng of *Clk*-luc (**a**) or *per*-luc (**b**); 50 ng of *renilla* luciferase) and expression vectors for V5-tagged PKA (5 ng) and HA-tagged CRTC (0, 50 or 250 ng). Dual luciferase reporter assays were performed 44 hours after transfection. Firefly luciferase activity was first normalized to that of *renilla* luciferase activity. Relative fold-activation was then calculated relative to baseline luciferase activity in the absence of any effectors. Data represent the average from three independent experiments. Error bars indicate SEM.

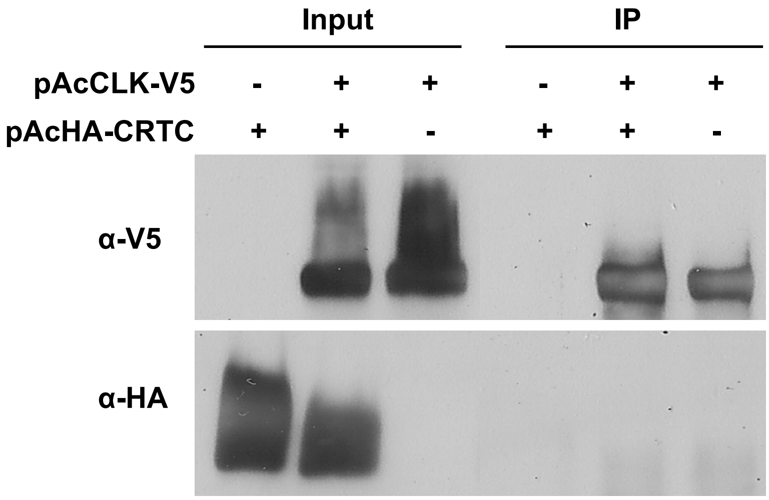

Supplementary Figure 11. CRTC does not physically interact with CLK
S2 cells were transfected with expression vectors for V5-tagged CLK and HA-tagged CRTC proteins. At approximately 44 hours after transfection, soluble extracts from transfected cells were immunoprecipitated with an anti-V5 antibody. Immunoprecipitated proteins were immunoblotted with mouse anti-V5 (top) and mouse anti-HA (bottom) antibodies, respectively. INPUT, approximately 5% of soluble extracts used for immunoprecipitation.

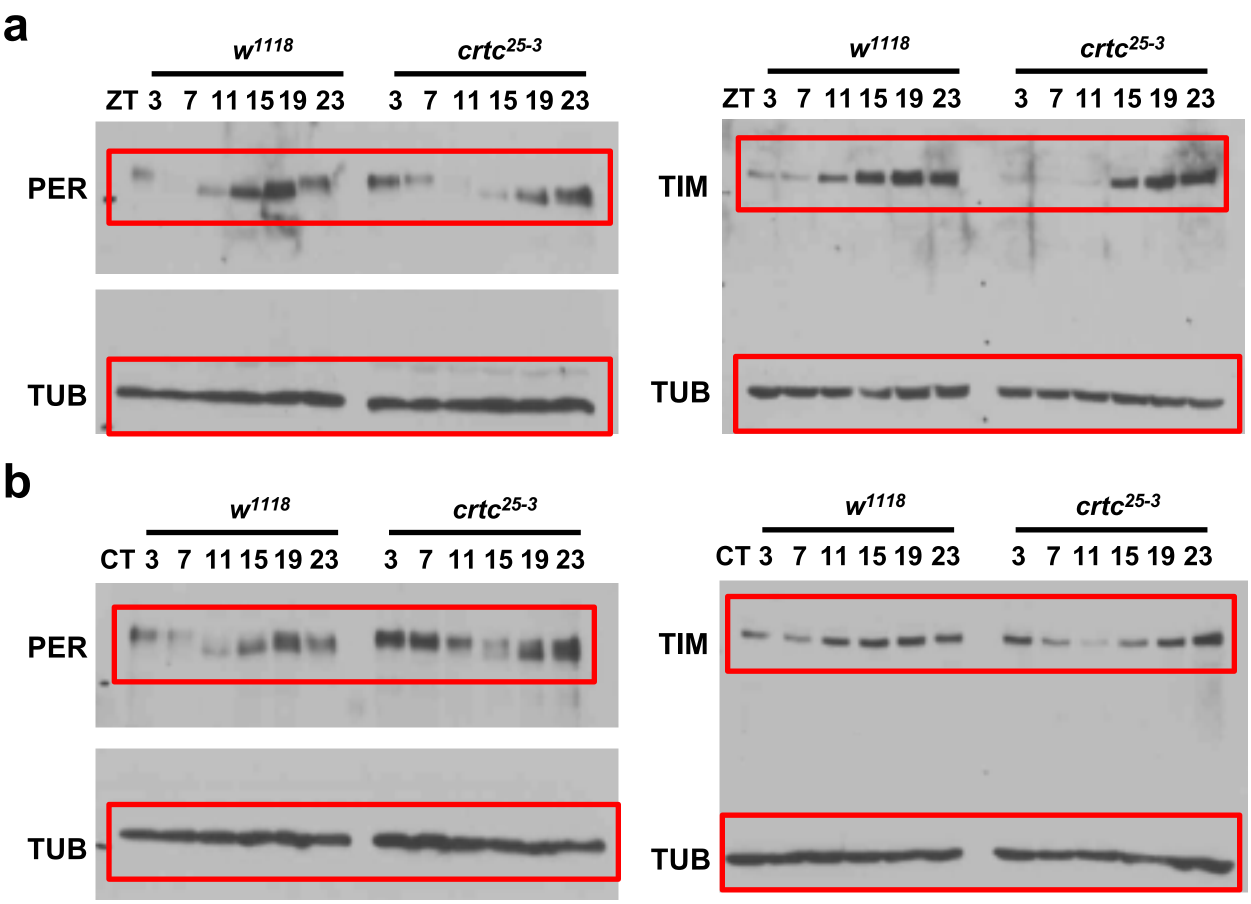

Supplementary Figure 12. *crtc* mutation phase-delays circadian expression of PER and TIM proteins in adult fly heads

Head extracts were prepared from flies harvested during LD cycle (**a**) or the first DD cycle (**b**) and immunoblotted with anti-PER, anti-TIM and anti-TUBULIN (TUB, loading control) antibodies under the same experimental conditions. Red rectangles include specific protein bands detected by each antibody in the full-length blot images. The cropped blot images were shown in Fig. 3b. ZT, zeitgeber time in LD cycles; CT, circadian time in DD cycles.

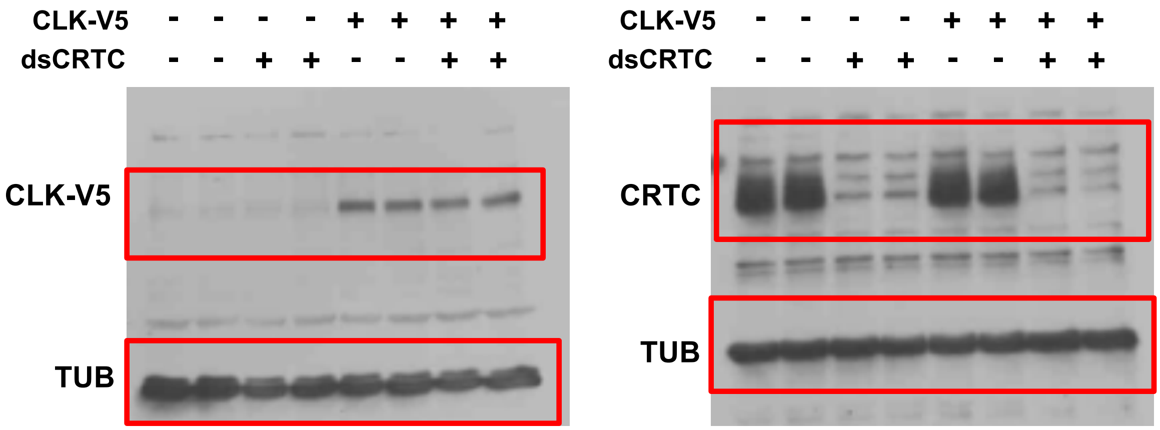

Supplementary Figure 13. dsCRTC specifically depletes endogenous CRTC but not V5-tagged CLK proteins in transfected S2 cells.
S2 cells were treated with 10 μg of dsEGFP or dsCRTC for 2 days. Where indicated, S2 cells were transfected with 10 ng of V5-tagged CLK expression vector. Cell extracts were prepared 2 days after transfection, resolved by SDS-PAGE and immunoblotted for each protein under the same experimental conditions. Red rectangles include specific protein bands detected by each antibody in the full-length blot images. The cropped blot images were shown in Fig. 5d.

Supplementary Table 1. Male flies homozygous or trans-heterozygous for a *crtc*-null mutation have weak but long-period locomotor rhythms

Genotype	n[a]	%R[b]	Period ± SEM (hr)	Power[c] ± SEM	Power[c] ± SEM of Rhythmic flies
w^{1118}	32	100	24.26 ± 0.05	112.58 ± 6.78	112.58 ± 6.78
$crtc^{25\text{-}3}$/+	30	90	23.68 ± 0.06	71.31 ± 8.82	78.47 ± 8.76
$crtc^{25\text{-}3}$	88	44	27.78*** ± 0.72	10.06*** ± 1.01	18.76*** ± 1.20
Df(3L)ED4710/+	27	100	23.90 ± 0.09	75.02 ± 5.43	75.02 ± 5.43
Df(3L)ED4710/$crtc^{25\text{-}3}$	39	48	28.36*** ± 1.20	10.21*** ± 1.17	16.49*** ± 1.06
Df(3L)BSC415/+	31	100	23.74 ± 0.15	90.03 ± 6.95	90.03 ± 6.95
Df(3L)BSC415/$crtc^{25\text{-}3}$	26	88	28.27*** ± 1.08	18.48*** ± 1.67	20.32*** ± 1.47

a n indicates number of flies analyzed

b %R indicates percent flies with detectable rhythmicity (P-S>10)

c Power is a measure of rhythmic strength

****P*<0.001 to wild-type (w^{1118}) and all heterozygous controls as determined by one-way ANOVA, Tukey post hoc test.

Supplementary Table 2. Enriched food does not rescue poor rhythmicity in DD locomotor behaviors of *crtc* mutants

Genotype		n[a]	%R[b]	Period ± SEM (hr)	Power[c] ± SEM	Power[c] ± SEM of Rhythmic flies
w^{1118}	5% sucrose	30	96	24.12 ± 0.10	110.93 ± 8.20	114.42 ± 7.68
w^{1118}	10% sucrose	31	100	24.08 ± 0.10	144.43 ± 5.92	144.43 ± 5.92
w^{1118}	Corn-meal food	29	100	24.13 ± 0.11	135.34 ± 8.46	135.34 ± 8.46
$crtc^{25\text{-}3}$	5% sucrose	22	86	29.78*** ± 1.00	19.72*** ± 2.34	21.67*** ± 2.41
$crtc^{25\text{-}3}$	10% sucrose	31	74	27.21* ± 1.22	15.23*** ± 1.47	19.06*** ± 1.12
$crtc^{25\text{-}3}$	Corn-meal food	28	60	27.61* ± 1.27	15.02*** ± 2.00	21.29*** ± 2.12

a n indicates number of flies analyzed
b %R indicates percent flies with detectable rhythmicity (P-S>10)
c Power is a measure of rhythmic strength
*P<0.05, ***P<0.001 to wild-type (w^{1118}) on each food as determined by Student's *t*-test

Supplementary Table 3. CRTC depletion in circadian pacemaker neurons leads to arrhythmic circadian behaviors

Genotype	n[a]	%R[b]	Period ± SEM (hr)	Power[c] ± SEM	Power[c] ± SEM of Rhythmic flies
UAS-*crtc* RNAi/+	27	96.2	23.55 ± 0.06	90.22 ± 10.04	93.67 ± 9.80
*Pdf*G4>UAS-DCR2/+	40	100	24.36 ± 0.06	121.20 ± 10.36	121.20 ± 10.36
*Pdf*G4>UAS-DCR2/+; UAS-*crtc* RNAi/+	32	81	23.86 ± 0.11	44.86** ± 6.74	53.82* ± 7.22
*tim*G4/+; UAS-DCR2/+	26	100	23.82 ± 0.09	137.49 ± 9.95	137.49 ± 9.95
*tim*G4/+; UAS-DCR2/UAS-*crtc* RNAi	32	46	29.07*** ± 0.85	18.98*** ± 1.80	21.37*** ± 1.59

a n indicates number of flies analyzed

b %R indicates percent flies with detectable rhythmicity (P-S>10)

c Power is a measure of rhythmic strength

*P<0.05, **P<0.01, ***P<0.001 to controls heterozygous for Gal4>DCR2 or UAS-*crtc* RNAi as determined by one-way ANOVA, Tukey post hoc test

Supplementary Table 4. SIK2 overexpression in circadian pacemaker neurons causes weak but long-period rhythms in free-running locomotor behaviors

Genotype	n[a]	%R[b]	Period ± SEM (hr)	Power[c] ± SEM	Power[c] ± SEM of Rhythmic flies
UAS-SIK/+	32	100	24.79 ± 0.08	113.53 ± 5.93	113.53 ± 5.93
*Pdf*G4/UAS-SIK	28	85	27.25* ± 0.72	25.89*** ± 3.80	29.90*** ± 3.86
*tim*G4/UAS-SIK	46	78	27.33* ± 0.93	16.86*** ± 1.61	20.56*** ± 1.55
*Pdf*G4/+	24	100	24.43 ± 0.04	101.74 ± 7.23	101.74 ± 7.23
*tim*G4/+	32	100	24.96 ± 0.04	128.33 ± 6.58	128.33 ± 6.58

a n indicates number of flies analyzed

b %R indicates percent flies with detectable rhythmicity (P-S>10)

c Power is a measure of rhythmic strength

**P*<0.05, ****P*<0.001 to controls heterozygous for Gal4 or UAS transgene as determined by one-way ANOVA, Tukey post hoc test

Supplementary Table 5. CRTC overexpression in PDF neurons partially rescues circadian behaviors in *crtc* mutant flies

Genotype	n[a]	%R[b]	Period ± SEM (hr)	Power[c] ± SEM	Power[c] ± SEM of Rhythmic flies
*Pdf*G4/+	24	100	24.43 ± 0.04	101.74 ± 7.23	101.74 ± 7.23
UAS-CRTC/+	31	96	23.55 ± 0.06	82.9 ± 8.48	85.41 ± 8.52
*Pdf*G4/UAS-CRTC	29	65	27.57* ± 0.83	20.13*** ± 3.24	29.13*** ± 3.41
*Pdf*G4/+; $crtc^{25\text{-}3}$	46	47	$27.52^{\#}$ ± 0.80	$9.94^{\#\#\#}$ ± 1.57	$18.63^{\#\#\#}$ ± 1.96
UAS-CRTC/+; $crtc^{25\text{-}3}$	73	61	$28.22^{\#\#\#}$ ± 0.79	$12.92^{\#\#\#}$ ± 1.02	$18.89^{\#\#\#}$ ± 0.73
*Pdf*G4/UAS-CRTC; $crtc^{25\text{-}3}$	93	86	$24.51^{**,\#\#}$ ± 0.24	$43.97^{***,\#\#\#}$ ± 3.53	$50.49^{***,\#}$ ± 3.60

a n indicates number of flies analyzed
b %R indicates percent flies with detectable rhythmicity (P-S>10)
c Power is a measure of rhythmic strength
**P*<0.05, ***P*<0.01, ****P*<0.001 to controls heterozygous for Gal4 or UAS transgene in wild-type or *crtc* mutant backgrounds; $^{\#}P<0.05$, $^{\#\#}P<0.01$, and $^{\#\#\#}P<0.001$ to the transgenic controls in wild-type as determined by one-way ANOVA, Tukey post hoc test

Supplementary Table 6. Conditional CRTC depletion or overexpression in PDF neurons of adult flies is sufficient to affect circadian behaviors

Genotype	n[a]	%R[b]	Period ± SEM (hr)	Power[c] ± SEM	Power[c] ± SEM of Rhythmic flies
*Pdf*GS/+ (EtOH)[d]	49	100	23.84 ± 0.05	136.32 ± 6.68	136.32 ± 6.68
*Pdf*GS/+ (RU486)[d]	52	98	24.86 ± 0.07	148.18 ± 5.25	151.02 ± 4.51
UAS-CRTC RNAi/+ (EtOH)	27	100	23.64 ± 0.04	134.18 ± 6.77	134.18 ± 6.77
UAS-CRTC RNAi/+ (RU486)	29	100	23.70 ± 0.06	133.25 ± 6.79	133.25 ± 6.79
UAS-CRTC/+ (EtOH)	31	96	23.60 ± 0.06	135.58 ± 6.87	139.92 ± 5.50
UAS-CRTC/+ (RU486)	32	100	23.67 ± 0.06	131.26 ± 6.34	131.26 ± 6.34
*Pdf*GS/UAS-CRTC RNAi (EtOH)	54	98	23.48 ± 0.02	95.12 ± 6.85	96.76 ± 6.78
*Pdf*GS/UAS-CRTC RNAi (RU486)	58	87	25.79** ± 0.30	40.66*** ± 4.28	45.68*** ± 4.43
*Pdf*GS/UAS-CRTC (EtOH)	52	100	23.71 ± 0.20	100.19 ± 6.40	100.19 ± 6.40
*Pdf*GS/UAS-CRTC (RU486)	61	86	23.58 ± 0.25	32.38*** ± 2.80	36.31*** ± 2.85

a n indicates number of flies analyzed

b %R indicates percent flies with detectable rhythmicity (P-S>10)

c Power is a measure of rhythmic strength

d circadian behaviors were monitored on fly foods containing either 1% ethanol (EtOH) as a vehicle control or 125 μM RU486

**P<0.01, ***P<0.001 compared to ethanol-fed controls for the Gal4/UAS trans-heterozygotes or all RU486-fed controls heterozygous for Gal4 or UAS transgene as determined by one-way ANOVA, Tukey post hoc test.

Supplementary Table 7. TIM overexpression in PDF neurons partially rescues circadian behaviors in *crtc* mutant flies

Genotype	n[a]	%R[b]	Period ± SEM (hr)	Power[c] ± SEM	Power[c] ± SEM of Rhythmic flies
UAS-*tim*/+	15	93	23.50 ± 0.00	120.67 ± 14.54	129.29 ± 12.58
UAS-*Pdp*/+	27	100	23.61 ± 0.06	150.36 ± 7.91	150.36 ± 7.91
UAS-*cry*/+	15	100	23.50 ± 0.00	182.04 ± 6.98	182.04 ± 6.98
*Pdf*G4/+	24	100	24.43 ± 0.04	101.74 ± 7.23	101.74 ± 7.23
*Pdf*G4/UAS-*tim*	19	100	23.81 ± 0.22	88.50 ± 9.40	88.50 ± 9.40
*Pdf*G4/UAS-*Pdp*	26	96	24.30 ± 0.41	88.16 ± 8.58	91.45 ± 8.25
*Pdf*G4/UAS-*cry*	16	100	23.56 ± 0.06	130.78 ± 10.95	130.78 ± 10.95
UAS-*tim*/+; $crtc^{25\text{-}3}$	21	57	$30.25^{\#\#\#}$ ± 0.66	$12.79^{\#\#\#}$ ± 1.83	$18.65^{\#\#\#}$ ± 1.64
UAS-*Pdp*/+; $crtc^{25\text{-}3}$	38	52	$27.92^{\#\#\#}$ ± 0.67	$11.57^{\#\#\#}$ ± 1.84	$16.54^{\#\#\#}$ ± 1.97
UAS-*cry*/+; $crtc^{25\text{-}3}$	27	55	$27.69^{\#\#}$ ± 0.94	$12.85^{\#\#\#}$ ± 1.79	$19.37^{\#\#\#}$ ± 1.83
*Pdf*G4/+; $crtc^{25\text{-}3}$	46	47	$27.52^{\#\#}$ ± 0.80	$9.94^{\#\#\#}$ ± 1.57	$18.63^{\#\#\#}$ ± 1.96
*Pdf*G4/UAS-*tim*; $crtc^{25\text{-}3}$	57	72	24.07*** ± 0.32	$35.15^{**,\#\#\#}$ ± 4.70	$47.87^{*,\#\#\#}$ ± 5.35
*Pdf*G4/UAS-*Pdp*; $crtc^{25\text{-}3}$	45	55	24.19*** ± 0.36	$16.05^{\#\#\#}$ ± 2.24	$19.90^{\#\#\#}$ ± 1.67
*Pdf*G4/UAS-*cry*; $crtc^{25\text{-}3}$	17	52	$27.72^{\#\#}$ ± 1.38	$11.94^{\#\#\#}$ ± 2.48	$19.26^{\#\#\#}$ ± 2.73

a n indicates number of flies analyzed
b %R indicates percent flies with detectable rhythmicity (P-S>10)
c Power is a measure of rhythmic strength
*P<0.05, **P<0.01 ***P<0.001 to controls heterozygous for Gal4 or UAS transgene in wild-type or *crtc* mutant backgrounds; $^{\#\#}P$<0.01, and $^{\#\#\#}P$<0.001 to the transgenic controls in wild-type as determined by one-way ANOVA, Tukey post hoc test
